# Identification of cow-level risk factors and associations of selected blood macro-minerals at parturition with dystocia and stillbirth in Holstein dairy cows

**DOI:** 10.1038/s41598-022-09928-w

**Published:** 2022-04-08

**Authors:** M. Bahrami-Yekdangi, G. R. Ghorbani, A. Sadeghi-Sefidmazgi, A. Mahnani, J. K. Drackley, M. H. Ghaffari

**Affiliations:** 1 Animal Science Research Institute of Iran, Agricultural Research, Education and Extension Organization (AREEO), 3146618361 Karaj, Iran; 2 Department of Animal Sciences, College of Agriculture, Isfahan University of Technology, PO Box, 84156-83111 Isfahan, Iran; 3 Department of Animal Sciences, University of Illinois, Urbana, IL 61801 USA; 4 Institute of Animal Science, University of Bonn, 53111 Bonn, Germany

**Keywords:** Physiology, Reproductive biology, Reproductive disorders

## Abstract

A deeper understanding of the risk factors for dystocia and stillbirth could help farmers make decisions about dairy cow management. The objectives of this study were to investigate cow-level risk factors associated with dystocia and stillbirth in a relatively large sample of dairy cows using multivariable linear regression models. The data consisted of 51,405 calving records of 14,546 Holstein cows from 3 dairy herds in Isfahan Province, Iran, collected between April 2011 and September 2017. To investigate the association between selected blood macro-minerals and the incidence of dystocia and stillbirth, blood samples were collected at the time of parturition from a random subset of these cows, which included 1311 animals. The incidence of dystocia and stillbirths averaged 14.7% and 4.3%, respectively. Results showed that calving year, calving season, dry period length, BCS, parity, calf sex, calf birth weight, twin status, and stillbirth were significantly associated with the incidence of dystocia. According to the Random Forest (RF) classifier, we found that dry period length, calf birth weight, and parity were the most important cow-level risk factors for the incidence of dystocia. Calving year, calving season, parity, twin status, dry period length, calf birth weight, calf sex, and dystocia were significantly associated with the incidence of stillbirths. The most important risk factors identified by the RF classifier for stillbirths were twin status, parity, dry period length, and calf birth weight. Also, interactions between the cow-level risk factors associated with dystocia and stillbirth were identified. The incidence of dystocia was associated with the interactions of twin status × calf birth weight and twin status × stillbirth. According to our analysis, the incidence of stillbirth is caused by interactions among several factors, such as twin status × length of dry period, twin status × calving season, and twin status × parity. The highest incidence of dystocia (21.3%) and stillbirths (5.4%) was observed in hypo-calcemic cows. In conclusion, twin status seems to be a determining factor for the incidence of stillbirths but not for dystocia. Finally, the results of this study may help the dairy industry make management decisions aimed at reducing dystocia and stillbirth rates.

## Introduction

Parturition is a critical time in the life cycle of dairy cows and calves. Dystocia and stillbirth are two common disorders that affect the production and reproductive performance of dairy cows^1–3^ and cause substantial economic consequences^3,4^. Dystocia is defined as difficult calving due to prolonged parturition or severe assisted parturition^5^. The incidence of dystocia in the United States ranges from 9.5 to 13.2% in primiparous cows and from 5.0 to 6.6% in multiparous cows from 1985 to 1996^6^. The prevalence of dystocia has been reported to be 8.2% in Iranian Holstein dairy cows^1^. According to Wall et al.^7^, 16.0% of cows in the United Kingdom require assistance during calving, and the prevalence of dystocia is estimated to be 1.5% to 22.0% worldwide^8^. The differences in dystocia incidence in the population of cows from different studies/countries are generally known^8,9^ and are highly dependent on calving management and the case definition of dystocia^10^. In the published literature, dystocia is often described as calving difficulty, level of assistance during calving, and ease of calving, ranging from routine to non-routine (i.e., fetal malpresentation or intervention during dystocia)^8^. Some reports classify dystocia using dystocia scores of 1–5, whereas other reports refer only to assisted calving, regardless of the type of assistance used (e.g., cesarean section or pulling a large calf). Therefore, the existence of an international agreement on the definition of dystocia would make studies more comparable.

Stillbirth is defined as the death of a calf that occurs after at least 260 days of gestation just before, during, or within 24 to 48 h after parturition^11,12^. Stillbirths often result in impaired reproductive and milk production as well as dam survival^13–15^ so economic losses from stillbirths are not limited to the value of the stillborn calf^15^. In a study that included 104,572 calving events from 16 dairy herds in Iran, the prevalence of stillbirths was reported to range from 2.9 to 9.8% (average 4.9%)^16^. Recently, Mahnani et al.,^3^ reported that the incidence of stillbirths in Iranian Holstein cows was 4.2% (range 3.4% to 6.8%). In the cohort of Japanese Black cattle, Uematsu et al.,^17^ found that prematurity and excessive fetal weight, as well as primiparity and low temperatures in winter, were the risk factors for stillbirth and dystocia. There is evidence that stillbirths reduce 305-day milk production, increase the number of inseminations in primiparous cows, contribute to a higher culling rate due to lower milk production and poor reproductive performance, and reduce the probability of conception^18–22^.

Dystocia and stillbirth may be caused by either maternal, fetal factors, or a combination of both^8^. In most cases, dystocia is the result of abnormalities in fetal presentation, position, or posture, but it may also be caused by fetal oversize, pelvic abnormalities, or uterine inertia^10^. Fetopelvic disproportion is a primary cause of dystocia in heifers, while fetal maldispositions are a major cause in pluriparous cows^8^. Also, the prevalence of fetal malposition has been reported to range from 1.0 to 51.0% in beef cattle^23^. A previous study with data from several breeds found that cows with twin birth had a higher likelihood of dystocia and stillbirths than cows with single births^24^. The stillbirth rates in Holstein herds have previously been found to be between 3.2 and 5.4% for single births, and between 12.9 and 15.7% for twin births^25,26^. Twin birth in dairy cows are generally undesirable, and estimated losses from twin calving in dairy cows range from $59 to $161 per twin pregnancy^27^. The association between twin status and the incidence of dystocia was inconsistent across studies. Some studies reported a higher incidence of dystocia^28^ while others reported a lower incidence of dystocia in single birth compared with twin birth^29^. Echternkamp and Gregory^29^ attributed the higher incidence of dystocia in twin birth compared with single birth (46.9% versus 20.6%) primarily to the abnormal presentation (37.0% versus 4.5%) of one or both twin calves at parturition. The occurrence of twin pregnancies in dairy cows with high milk production is most likely caused by multiple ovulations and low circulating levels of progesterone^30^.

Dystocia is often the primary cause but accounts for only about 50% of stillbirths^31^. As stated in a recent study^32^, control of the age of the first calving is an essential management tool to achieve lower dystocia risk and higher lactation performance in dairy cows. Twin pregnancy could increase the incidence of dystocia, the incidence of freemartinism, the overall risk of culling and perinatal mortality, and decrease calf birth weight, milk production, and cow fertility^27^.

Studies have demonstrated a relationship between metabolic profile and reproductive disorders^33,34^. According to Heuwieser et al.^35^ and Hudson et al.^36^, dystocia was associated with higher blood cortisol levels. In another study, Vannucchi et al.^37^ reported that blood glucose concentrations were higher in cows who experienced dystocia compared to cows with no dystocia (n = 10/group). A mineral imbalance has an important effect on the reproductive physiology of dairy cows^38,39^, resulting in reduced reproductive efficiency. However, few studies have investigated the relationship between blood macro-minerals and the incidence of stillbirths and dystocia in dairy cows. Cows with prolonged or delayed subclinical hypocalcemia are more susceptible to disease and produce less milk than cows with normal postpartum plasma calcium (Ca) concentrations^40,41^. A previous study reported that cows that experienced dystocia (n = 22) had lower plasma Ca concentrations 24 h after calving than their normal counterparts (n = 25)^42^. Nevertheless, the sample sizes of previous studies investigating the association between blood parameters during parturition, dystocia, and stillbirths were relatively small.

Using multivariable linear regression models, the objectives of this study were to identify cow-level risk factors associated with the incidence of dystocia and stillbirth in a relatively large sample of dairy cows. In addition to logistic regression analysis, we used feature importance in Random Forest (RF) classifier for identifying the most important cow-level risk factors that contribute most strongly to the incidence of dystocia and stillbirth. As a second objective, we examined selected macro-minerals in blood at the time of parturition in relation to dystocia and stillbirth in mineral-deficient vs. normal cows.

## Results

**Figure 1:**
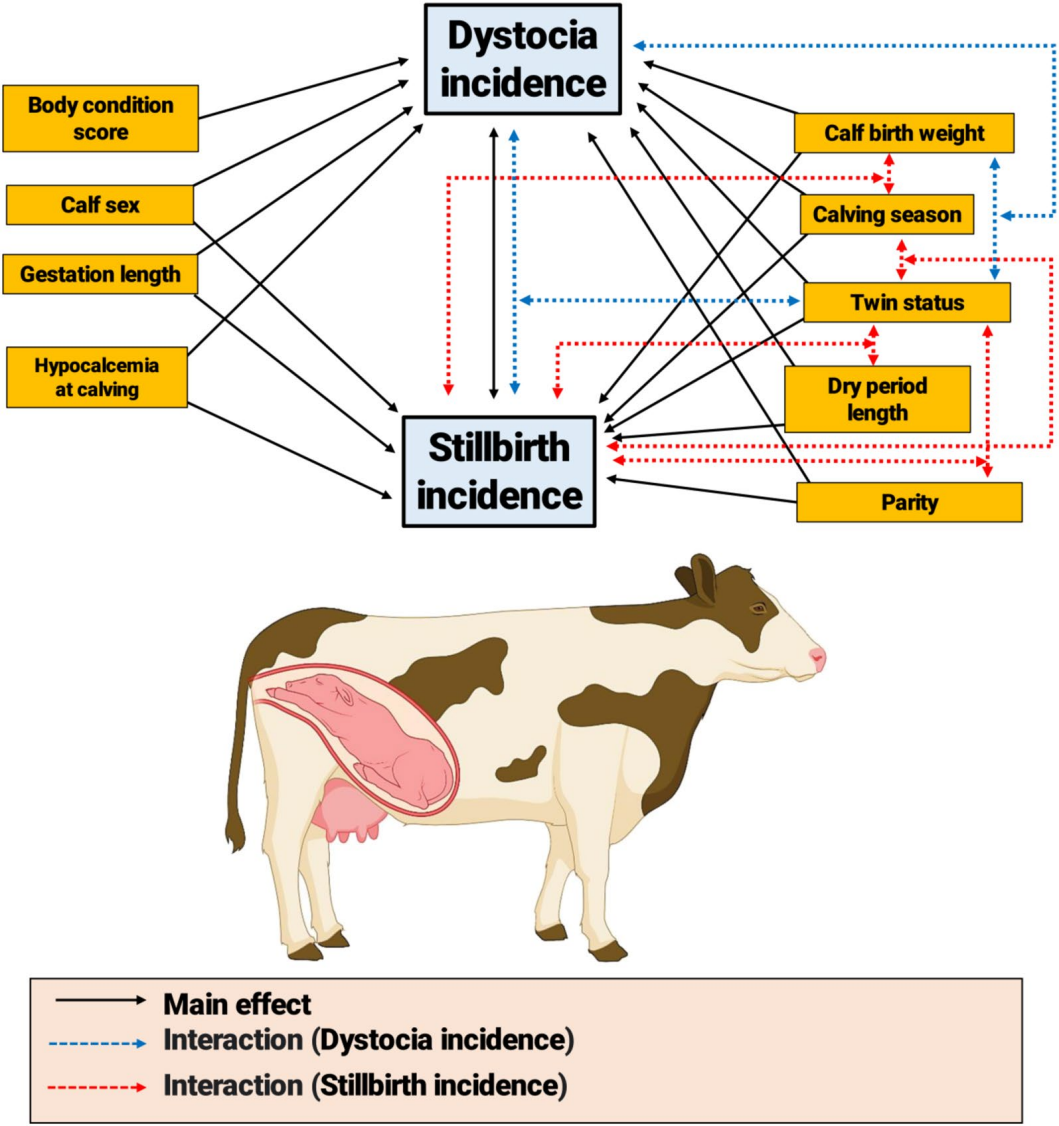
A causal diagram illustrating the main effects and interactions among cow-level factors associated with the incidence of dystocia and stillbirths using the results of multivariable logistic regression models. The Figure was designed by BioRender online software (https://app.biorender.com).

This study focused on large industrial dairy herds in Isfahan Province, Iran. A summary of the data is provided in Table 1. To ensure the integrity of the data and to avoid inconsistencies among the herds studied, traits were categorized based on their most common definitions, as shown in Table 2. Figure 1 shows a causal web illustrating the main effects and interactions among cow-level factors associated with the incidence of dystocia and stillbirths using the results of multivariable logistic regression models.

**Table 1:** Characteristics of the investigated herds.

Variable	Herds		
	1	2	3
Number of milking cows, head	4800	2500	3000
Average incidence of dystocia, %	12.0	22.5	14.5
Average incidence of stillbirth, %	4.29	8.23	2.16
Average incidence of twinning, %	2.50	5.50	3.80
Average gestation length, d	276	276	276
Average dry length, d	67	63	62
Average body condition score at calving	3.21	3.40	3.24
Calf birth weight, kg	41.90	40.72	40.23
Average milk yield, kg	42.00	41.80	41.90

**Table 2:** The definitions of the traits used in the study.

Dystocia	Any help (from only slight assistance to caesarean) provided to cows during their parturition. Recognized dystocia scores were 1 = no assistance, 2 = slight assistance, 3 = difficult calving (mechanical assistance), 4 = difficult calving (veterinary assistance), 5 = calving requiring caesarean. In the present study, dystocia scores of 1 or 2 were coded as easy calving, and scores of ≥ 3 were coded as dystocia
Stillbirth	A calf loss from days 260 to 48 h after calving
Body condition score (BCS)	Cows were assigned a score of 1 to 5, with a BCS of 1 considered as extremely thin and 5 as extremely fat. For this study, use was made of the dry cow score and the animals were assigned to 3 groups: (1) cows with BCS < 3.25, (2) those with BCS in the range of 3.25 to 3.75, and (3) those with BCS > 3.75
Dry period length	Based on their dry period length, the cows were classified into 4 groups: (1) those with dry periods < 50 days, (2) those with dry lengths between 51 and 65 days, (3) those with dry lengths from 66 to 100 days, and (4) those with dry lengths > 100 days
Calf birth weight	Calves weighing less than 15 kg and more than 70 kg were removed from the dataset. The remaining calves were classified into 7 groups: (1) those weighing < 35 kg, (2) those between 35.1 and 38 kg, (3) those between 38.1 and 40 kg, (4) those between 40.1 and 42 kg, (5) those between 42.1 and 44 kg, (6) those between 44.1 and 46 kg, and (7) those weighing > 46 kg

### Cow-level risk factors of dystocia

Calving number and percentage of stillbirths for variables included in the binary logistic regression model of the generalized linear mixed model for cow-level risk factors for dystocia incidence are shown in Table 3. Estimated odds ratios (OR) and 95% confidence levels (CI) of the risk factors are shown in Supplemental Table S1A-B. A mean dystocia incidence of 14.7% was determined. Dystocia incidence varied across calving years (*P* < 0.01). The lowest (13.7%) and highest (17.2%) dystocia incidence was recorded in calving years 2016 (OR = 1.00) and 2015 (OR = 1.34), respectively. The incidence of dystocia varied by season (within 10% significance threshold, *P* = 0.08), with the lowest and highest incidence of 14.3% and 15.9% recorded in summer (OR = 0.99) and winter (OR = 1.08), respectively. The incidence of dystocia was higher in cows of parity 1 (15.5%; referent) and > 4 (15.1%; OR = 0.86) than in the other parities (*P* < 0.01). An association was found between dry period length and dystocia incidence (*P* < 0.01). Cows with dry period lengths between 45 and 60 days (OR = 0.47) had the lowest incidence (10.8%). Dystocia incidence increased with increasing dry period length. Additionally, gestational length affected the incidence of dystocia (*P* < 0.01). The incidence of dystocia was higher for male (OR = 1.36) than female (referent) calves (17.6%: male vs. 11.7%: female; *P* < 0.01). The incidence of dystocia was higher for overconditioned cows (body condition score, BCS > 3.75; OR = 1.27) than other cows (*P* < 0.01).

**Table 3:** Calving number and percentage of stillbirths for variables included in the binary logistic regression model of the generalized linear mixed model for cow-level risk factors for dystocia incidence in Holstein dairy cows (n = 51,405).

Variable	Calving No	Dystocia%	*P*-value
**Calving year**			< 0.01
2011	6236	14.2	
2012	6775	13.9	
2013	6925	14.2	
2014	8215	15.1	
2015	8615	17.2	
2016	9640	13.7	
2017	4999	14.6	
**Calving season**			
Spring	11,114	14.6	0.08
Summer	15,687	14.3	
Autumn	13,498	14.5	
Winter	11,106	15.9	
**Parity**			< 0.01
1	31,093	15.5	
2	8944	12.9	
3	5378	13.5	
≥ 4	5990	15.1	
**Twin status**			< 0.01
Single birth	49,591	14.1	
Twin birth	1814	32.5	
Twin birth × Calf birth weight			< 0.01
Twin birth × Stillbirth			0.01
**Dry period length**			< 0.01
≤ 45	26,416	13.4	
46–60	16,056	10.8	
61–100	5957	14.8	
> 100	2976	18.7	
Gestation length		1.0	0.01
**Calf birth weight**			< 0.01
≤ 35	6806	13.8	
35.1–38	8424	11.6	
38.1–40	8335	11.6	
40.1–42	7092	11.8	
42.1–44	7085	13.6	
44.1–46	4960	16.0	
46 <	8703	24.3	
**Calf sex**			0.01
Female	25,504	11.8	
Male	25,901	17.6	
**Stillbirth**			< 0.01
No	49,191	14.0	
Yes	2214	31.8	
**BCS**			< 0.01
< 3.25	27,890	14.1	
3.25 -3.75	20,828	15.5	
> 3.75	2687	16.6	

**Figure 2:**
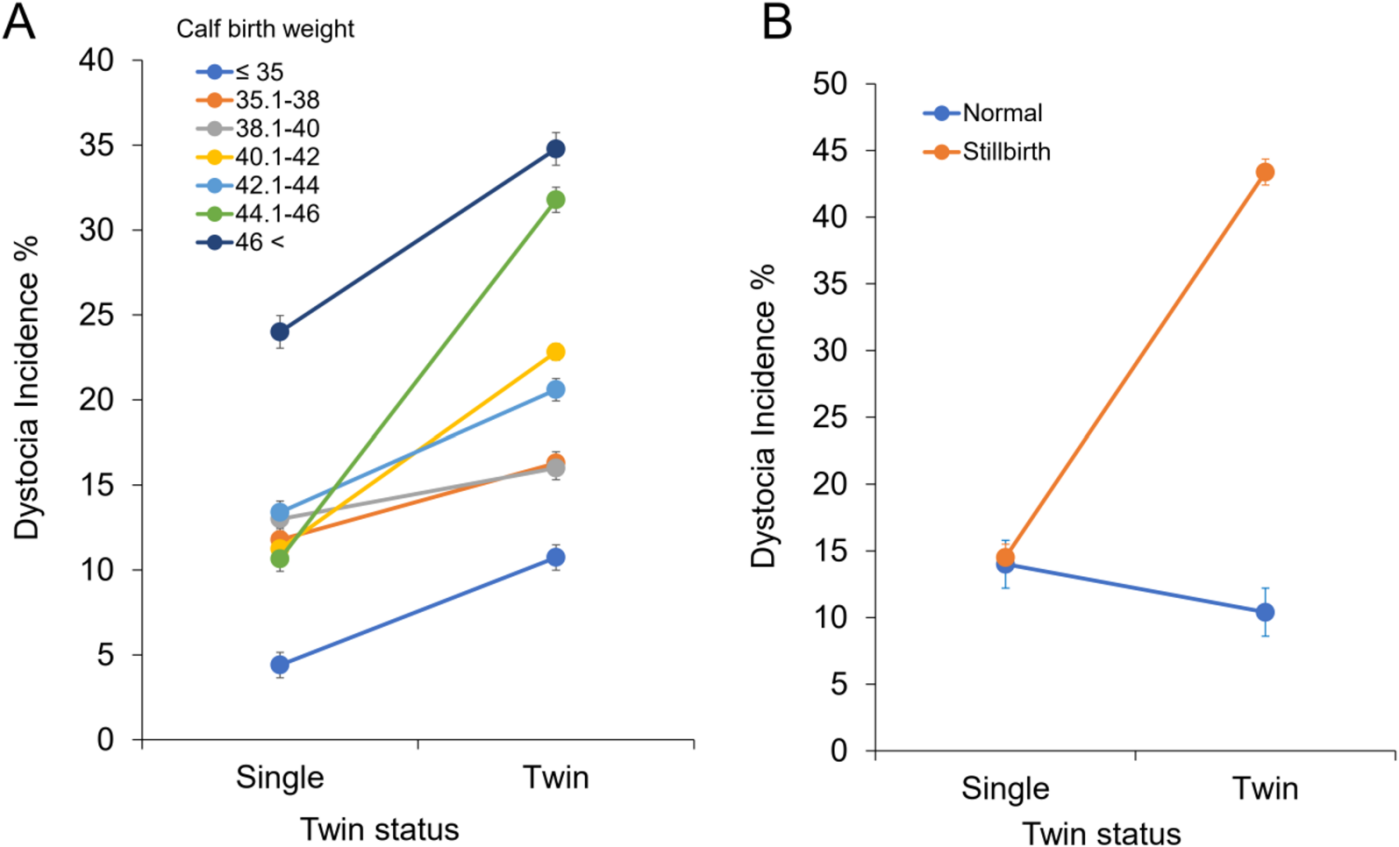
Interaction effects of twin status × calf birth weight (**A**) and twin status × stillbirth (**B**) were significantly associated with dystocia incidence.

Also, interaction effects of twin status × calf birth weight and twin status × stillbirth were significantly associated with dystocia incidence (Fig. 2, *P* < 0.01). When cows give birth to twins, calves weighing more than 44 kg at birth are more likely to have dystocia (Fig. 2A). The incidence of dystocia in cows that gave birth to stillborn calves was higher in twins than in singletons (Fig. 2B).

### Selected blood macro-minerals

Calving number and percentage of stillbirths for selected blood macro-minerals at parturition included in the binary logistic regression model of the generalized linear mixed model for dystocia incidence are shown in Table 4. Estimated OR and 95% CI of selected blood macro-minerals at the time of parturition that were associated with the incidence of dystocia are shown in Supplemental Table S2. Blood concentrations of Ca (*P* = 0.02) and P (within 10% significance threshold, *P* = 0.09) at the time of parturition that were associated with the incidence of dystocia. The highest incidence of dystocia was observed in cows with hypocalcemia (≤ 8 mg/dL) and hypophosphatemia (≤ 4 mg/dL) at the time of parturition. Blood concentrations of magnesium (Mg) at the time of parturition were not significantly associated with the incidence of dystocia.

**Table 4:** Calving number and percentage of stillbirths for selected blood macro-minerals at parturition included in the binary logistic regression model of the generalized linear mixed model for dystocia incidence in Holstein dairy cows (n = 1311).

Variable	Calving No	Dystocia%	*P*-value
**Calcium (mg/dl)**			0.02
Hypo (≤ 8)	435	21.3	
Normal (> 8.1)	876	17.2	
**Phosphorus (mg/dl)**			0.09
Hypo (≤ 4)	355	20.3	
Normal (> 4.1)	956	18.0	
**Magnesium (mg/dl)**			0.19
Hypo (≤ 2.5)	379	17.7	
Normal (> 2.51)	932	18.8	

Variance	Estimate	SE	
Residual variance	0.945	0.032	
Herd variance	0.019	0.051	

### Cow-level risk factors of stillbirth

Calving number and percentage of stillbirths for variables included in the binary logistic regression model of the generalized linear mixed model for cow-level risk factors for stillbirth incidence are shown in Table 5. Estimated OR and 95% CI of the risk factors for stillbirth incidence are shown in Supplemental Table S3A–D. A mean stillbirth incidence of 4.3% was determined. Stillbirth incidence varied across calving years (*P* < 0.01). The lowest (3.2%) and highest (5.3%) incidence of stillbirths was recorded in calving years 2016 (OR = 0.37) and 2012 (OR = 1.02), respectively. Additionally, gestational length affected the incidence of stillbirths (*P* < 0.01). The incidence of stillbirths was higher (*P* = 0.02) for males (OR = 1.16) than for female (referent) calves (4.1%: male calves vs. 4.5%: female calves. The incidence of stillbirths was higher with dystocia (*P* < 0.01; OR = 2.04) compared with non-assisted births (9.3% vs. 3.4%, respectively).

**Table 5:** Calving number and percentage of stillbirths for variables included in the binary logistic regression model of the generalized linear mixed model for cow-level risk factors for stillbirth incidence in Holstein dairy cows (n = 51,405).

Variable	Calving No	Stillbirth%	*P*-value
**Calving year**			< 0.01
2011	6236	4.63	
2012	6775	5.28	
2013	6925	5.09	
2014	8215	4.35	
2015	8615	4.36	
2016	9640	3.20	
2017	4999	3.42	
**Calving season**			
Spring	11,114	4.20	< 0.01
Summer	15,687	4.89	
Autumn	13,498	4.26	
Winter	11,106	3.57	
Calving season × Twin status			< 0.01
Calving season × Calf birth weight			< 0.01
**Parity**			< 0.01
1	31,093	3.34	
2	8944	5.75	
3	5378	5.93	
4 ≤	5990	5.34	
Parity × Twin status			< 0.01
**Twin status**			< 0.01
Single birth	49,591	2.00	
Twin birth	1814	6.70	
**Dry period length**			0.08
< 45 days	26,416	4.10	
46–60 days	16,056	4.44	
61–100 days	5957	4.44	
> 100 days	2976	5.30	
Dry period length × Twin status			< 0.01
Gestation length			< 0.01
**Calf birth weight**			< 0.01
≤ 35	6806	9.63	
35.1–38	8424	4.99	
38.1–40	8335	2.61	
40.1–42	7092	2.50	
42.1–44	7085	2.83	
44.1–46	4960	2.70	
46 <	8703	3.87	
**Calf sex**			0.02
Female	25,504	4.12	
Male	25,901	4.49	
**Dystocia**			< 0.01
No	43,810	3.44	
Yes	7595	9.26	

**Figure 3:**
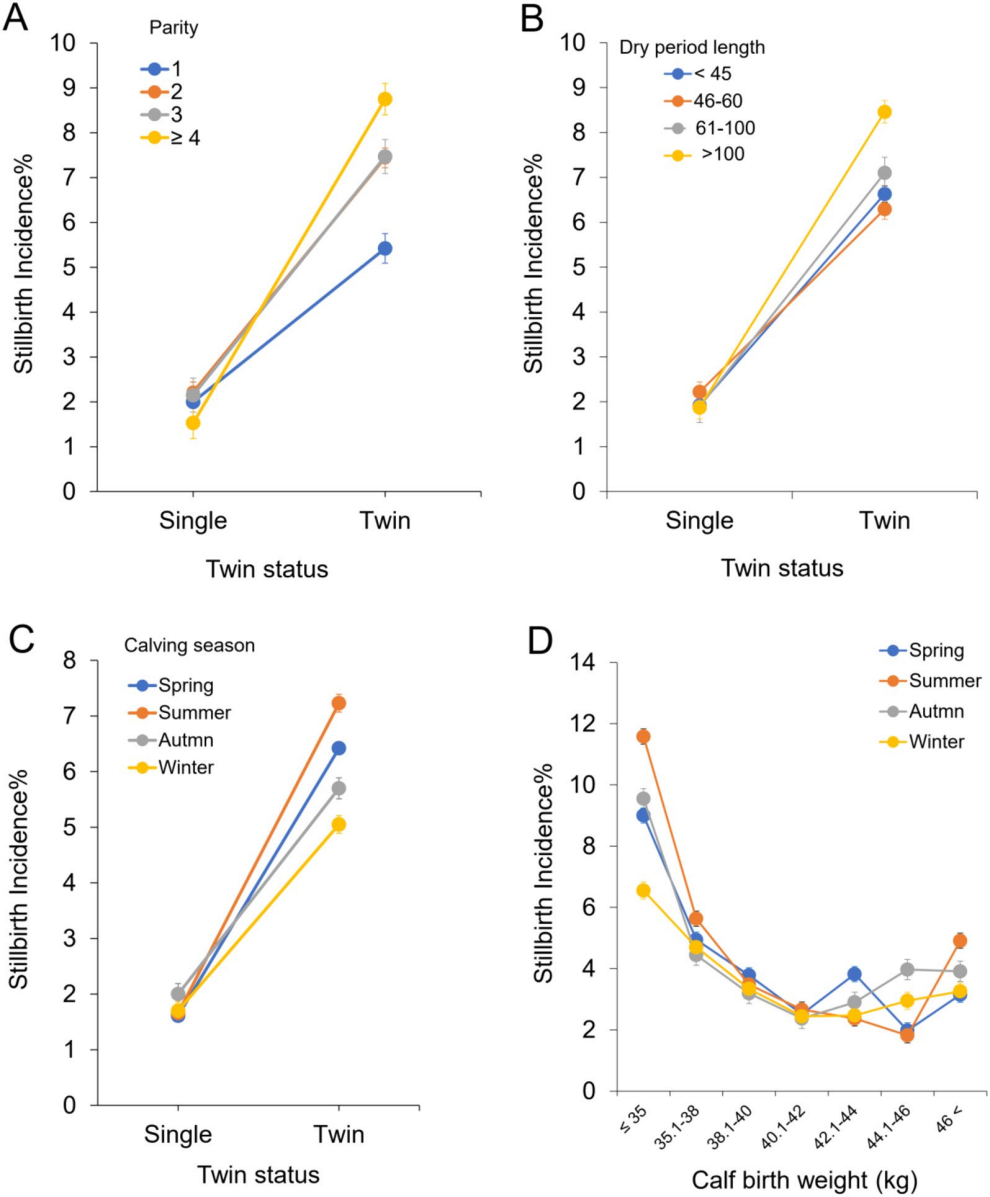
Interaction effects of parity × twin status (**A**), dry period length × twin status (**B**) and calving season × twin status (**C**) and calving season × calf birth weight (**D**) were significantly associated with the incidence of stillbirth.

Interaction effects of parity × twin status, dry period length × twin status, and calving time × twin status and calving time × calf birth weight were significantly associated with the incidence of stillbirths (*P* < 0.01; Fig. 3). In cows that gave birth to twins, the risk of stillbirth was greater for cows with parity > 4 than for other parities (Fig. 3A). Cows with a dry period > 100 days had a higher risk of stillbirth in cows that gave birth to twins (Fig. 3B). Cows calving in summer had a higher rate of stillbirth than in other seasons in cows giving birth to twins (Fig. 3C). In addition, calving in summer may increase the risk of stillbirth when calves are born with low birth weight (≤ 35 kg) (Fig. 3D).

### Selected blood macro-minerals

Calving number and percentage of stillbirths for selected blood macro-minerals at parturition included in the binary logistic regression model of the generalized linear mixed model for stillbirth incidence are shown in Table 6. Estimated OR and 95% CI of selected blood macro-minerals at the time of parturition that were associated the incidence of stillbirths are shown in Supplemental Table S4. Blood concentrations of Ca at the time of parturition that were associated with the incidence of stillbirths (*P* = 0.01). The highest incidence of stillbirths was observed in cows with hypocalcemia (≤ 8 mg/dL) at the time of parturition. Blood concentrations of P and Mg at parturition were not significantly associated with the incidence of stillbirths.

**Table 6:** Calving number and percentage of stillbirths for selected blood macro-minerals at parturition included in the binary logistic regression model of the generalized linear mixed model for stillbirth incidence in Holstein dairy cows (n = 1311).

Variable	Calving No	Stillbirth%	*P*-value
**Calcium (mg/dl)**			0.01
Hypo (≤ 8)	435	5.38	
Normal (> 8.1)	876	3.82	
**Phosphorus (mg/dl)**			0.35
Hypo (≤ 4)	355	4.27	
Normal (> 4.1)	956	4.04	
**Magnesium (mg/dl)**			0.88
Hypo (≤ 2.5)	379	4.38	
Normal (> 2.51)	932	4.11	

Variance	Estimate		
Residual variance	0.536		
Herd variance	0.031		

#### Random forest

**Figure 4:**
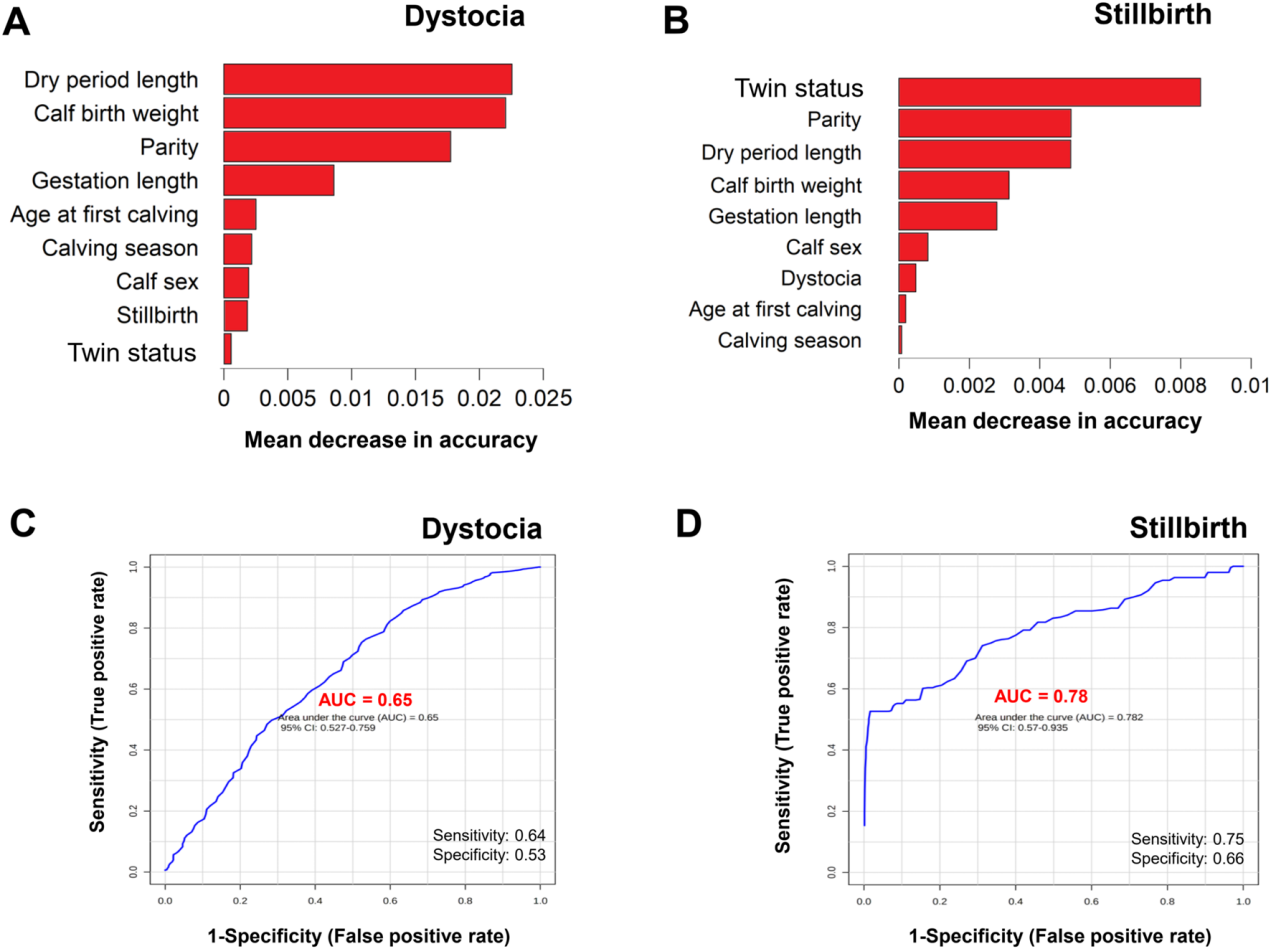
Important calf-level risk factors (features) were identified for (**A**) dystocia and (**B**) stillbirth by random forest classification in ascending order based on the mean decrease accuracy (n = 51,405). The ROC (receiver operating characteristic) curves for the random forest model for dystocia (**C**) and stillbirth (**D**).

Figure 4 shows the cow-level risk factors identified by RF for dystocia (Fig. 4A) and stillbirth (Fig. 4B), ranked by mean decrease accuracy. Figure 4C and D illustrate the ROC (receiver operating characteristic) curves for dystocia and stillbirth, respectively. The area under the ROC curve (AUC) was 0.65 (95% CI 0.53–0.76) and 0.78 (95% CI 0.57–0.94) for dystocia and stillbirth, respectively. Based on RF classification models, there were sensitivity and specificity values of 64% and 53% (for dystocia) and 75% and 66% (for stillbirth), respectively. The highest-ranking factors for dystocia were dry period length, calf birth weight, and parity. The highest-ranking factors for stillbirth were twin status, parity, dry period length, and calf birth weight.

## Discussion

### Cow-level risk factors associated with dystocia and stillbirth

A total of 51,405 calving records of 14,546 cows from 3 dairy herds in Isfahan Province (Iran) were evaluated for potential cow-level risk factors associated with dystocia and stillbirths. The incidences of dystocia and stillbirth averaged 14.7% and 4.3%, respectively. The results of the final multivariable logistic regression models were used in this study to test for the main effects and interaction effects of cow-level risk factors associated with the incidence of dystocia and stillbirth in a relatively large sample of dairy cows. The results showed that calving year, calving season, dry period length, BCS, parity, calf sex, calf birth weight, twin status, and stillbirth were significantly associated with dystocia incidence. In addition to logistic regression analysis, we used feature importance in RF classifier for identifying the most important cow-level risk factors that contribute most strongly to the incidence of dystocia and stillbirth. Feature importance in the RF classifier is an effective method to reduce the number of input variables to the most important ones and to maintain the interpretability of the final model^43^. Using RF, we found that dry period length, calf birth weight, and parity were the most important cow-level risk factors for the incidence of dystocia. Calving year, calving season, parity, twin status, dry period length, calf birth weight, calf sex, and dystocia were significantly associated with stillbirth incidence. The most important risk factors identified by the RF for stillbirths were twin status, parity, dry period length, and birth weight of the calf.

As reported in this study, the incidence of dystocia was lowest in summer and highest in winter, at 14.3% and 15.9%, respectively. When comparing winter and summer calvings, dystocia was more likely to occur during the winter and less likely during the summer, perhaps because in colder winters, blood flow to the uterus increases, leading to a heavier calf^44–46^. Despite this, we found no interaction between calving season, calf birth weight, and dystocia incidence in the present study. Our results are consistent with those obtained previously by Gaafar et al.,^47^ who found that dystocia was most prevalent in winter and least prevalent in summer in dairy Friesian cows. In regards to the factors mentioned above, dry period length has been associated with the risk of dystocia. We found that a longer dry period was associated with a higher incidence of dystocia, and cows with a long dry period of more than 100 days had the highest incidence, whereas cows with a shorter dry period between 46 and 60 days had the lowest incidence. Similar results were reported by Atashi et al.^2^ which reported a more frequent incidence of dystocia in cows with a dry period > 60 days compared to cows with a shorter dry period. In addition, Enevoldsen and Sørensen^48^, found that dystocia was more likely in cows with long dry period (10 weeks) lengths than in cows with a dry period of 4 to 7 weeks. Consistent with this, Barkema et al.,^49^ reported that cows with a long dry period were more likely to have an increased risk of cesarean section. Cows with long dry periods gain excessive body fat during the extended dry period as the cows do not regulate their feed intake according to their physiological needs^50^. Cows which experience dystocia are more likely to have a higher body condition (more body fat) as a result of their prolonged dry periods. Roche et al.^51^ reported an increased risk of metabolic disorders associated with an excess body condition at calving. Besides over-conditioning, the long dry period may result in altered Ca metabolism^52^.

The higher incidence of dystocia in primiparous cows (parity 1) as compared to multiparous cows (parities 2 and 3) observed in the current study, agrees with previous studies^53,54^. The higher risk of dystocia in primiparous than multiparous cows may be due to feto-pelvic disproportion and abnormal fetal position, and uterine inertia in primiparous cows^8^. In some cases, dystocia may result from the disproportionate size of the fetus with respect to the pelvic area of the dam^55^ and may be influenced by the birth weight of the calf. In this study, male calves were more likely to cause dystocia than female calves, with is in line with previous reports^1,56^. We observed that male calves with a birth weight over 42.7 ± 6 kg (mean ± SD) were larger than female calves with less than 39.5 ± 5 kg (mean ± SD). In this study, a higher risk of dystocia was observed in cows that gave birth to male calves than in cows that gave birth to female calves. A part of this could be due to the fact that male calves are heavier^1,56^ and have larger dimensions^57^ than female calves, or it could also be due to other factors.

There is evidence that male calves are at risk for several reproductive disorders, including dystocia^1^ and stillbirths^15^, which have been associated with increased risk of mortality in dairy cows^15,58^. The percentage of male and female calves in this study was 50.5% and 49.5%, respectively, and male calves were more likely to be stillborn, which is consistent with the results of Maltecca et al.^59^ that male calves have a higher risk of stillbirth than female calves. According to a recent study, the stillbirth rate for male calves (7.7%) was more than twice as high as that of female calves (3.7%)^60^. Mellado et al.^61^ found that calves with a birth weight of < 35 kg had the highest probability of stillbirth, which is in line with our findings.

Gestation length, the period from conception to calving, has a significant impact on the breeding and performance of cattle^62^. Stillbirths are associated with both longer and shorter gestation periods^63^. According to Nogalsk and Piwczyński^62^, the optimal gestation length was found in the range of 275–277 days based on the incidence of dystocia and stillbirth. In a study of more than 4000 Friesian cows, Johanson and Berger^45^ found that 282 days was the optimal gestation length that minimizes the risk of stillbirth. Deviation from an optimal value for gestation length has resulted in increased stillbirth rates^62^. The average gestation length was 274 ± 5 days for cows with twin birth and 277 ± 5 days for cows with single births in the current study. Previous studies reported that cows calving twins were more likely to have stillbirths than cow calving singletons, probably due to shorter gestational periods and higher incidences of dystocia^64,65^. Early-born calves are more likely to die of oxygen deprivation (birth asphyxia) within 2 days, which may contribute to the higher stillbirth rate among twins. Therefore, cows that suffer dystocia when they calve twins are at increased risk for stillbirths.

### Interactions

The interactions among multiple risk factors that determine the incidence of dystocia and stillbirth were evaluated and illustrated (Fig. 1). According to our results, the incidence of dystocia was associated with the interactions of twin status × calf birth weight and twin status × stillbirths. In the current study, calving calves that weigh more than 44 kg at birth may increase the risk of dystocia if the cows give birth to twins compared with singletons. This interaction can be explained by the fact that twins with higher birth weight (> 44 kg) have a more negative effect on feto-pelvic disproportion than single births. Dystocia is strongly associated with the ratio between the pelvic area of the dam and the birth weight of the calf^55,66^. Fetopelvic disproportion is caused by an incompatibility between the size of the fetus and the size of the maternal pelvis, which may result from one or a combination of factors^67^. Therefore, a heavy twin calf may have a harder time going through a smaller pelvic area than a light twin calf. There are other factors, such as over-expansion of the uterus due to the weight of the twins and their placenta, and impaired metabolism due to less space for the rumen in the abdomen, that could also affect the ease of calving in cows with twin fetuses compared to cows with single births.

The incidence of dystocia was associated with the interaction between twin status × stillbirths. The current study found that the incidence of dystocia in cows giving birth to stillborn calves increased in twins compared to singletons. There is a positive correlation between twin birth and stillbirths^68^. This interaction can be explained by the fact that cows with twins are more likely to have inadequate abdominal contractions and to deliver calves in an abnormal position, presentation, and posture^69^, which results in a higher risk of stillbirth. The interaction between twin status and dry period length was significantly associated with the incidence of stillbirths in the current study. According to the current study, the risk of dystocia was greater in cows with a dry period > 100 days when cows calving twins than those calving singletons. This interaction can be explained by the fact that a longer dry period means that cows may be exposed to anabolic influences for a longer period, increasing the likelihood of over-conditioning at calving^70^, higher calf birth weight, and stillbirth risk in twin calves. The interaction between twin status and parity was significantly associated with the incidence of stillbirths in the current study. In dairy cows, twinning increases with increasing parity, ranging from 1% at first parity to nearly 10% at later parities^27^. In this study, there was a greater incidence for twin calves to be stillborn when their dam at their fourth or greater parities (≥ 4). This interaction can be explained by the fact that the occurrence of twins in dairy cows increases with age (~ 4 to 30% with primiparous and multiparous cows), which is due to the increased occurrence of multiple ovulations^71,72^. One explanation is that due to a higher embryonic mortality rate in twin pregnancies^73^, a twin pregnancy in older cows has a lower probability of survival and is more likely to result in embryonic loss or stillbirth than in younger cows^71^. This could be because one embryo prevents the other from settling in the uterus, depriving it of nutrients and converting the pregnancy from twins to singletons, as has been documented in mares^74^.

The interaction between twin status and calving season was significantly associated with the incidence of stillbirths in the current study. Under conditions of heat stress, dams and their offspring show altered physiological responses, tissue development, metabolism, and immunity^75^. In utero hyperthermia negatively affects fetal growth and postnatal physiology of offspring, such as immune function and metabolic adaptation^76–78^. Cows that suffered from severe heat stress prenatally and at birth (THI > 83 units) were 1.3 more likely to stillbirth than cows with lower heat stress^61^. In the current study, a higher rate of stillbirths was observed in cows calving twins in summer than in other seasons. This can be explained by the fact that dams exposed to heat stress in late gestation delivered calves with lower birth weights, indicating impaired fetal development^75,79,80^. Therefore, heat stress in late gestation leads to intrauterine growth restriction and can decrease placental weight^81,82^, which may explain part of the lower birth weight due to in utero hyperthermia in summer than in winter in stillborn calves in cows that gave birth to twins.

### Associations of blood macro-minerals at parturition with dystocia and stillbirth

We also compared dystocia and stillbirth in cows with macro-minerals deficiency and normal cows at the time of parturition. Normal blood Ca level in dairy cows is between 2.1 and 2.5 mmol/L (8.5 and 10 mg/dL)^83^. Based on available data, the highest incidence of dystocia (21.3%) and stillbirths (5.4%) was observed in the group of cows with plasma Ca concentrations less than 8 mg/dL at the time of parturition, which is considered hypocalcemia. These results are consistent with previous studies in which clinical hypocalcemia was associated with periparturient disorders such as dystocia^84^. Benzaquen et al.^42^ reported that dystocic cows had lower plasma Ca concentrations but increased plasma haptoglobin 24 h after calving compared with cows with normal parturition. Dairy cows have clinical hypocalcemia (milk fever) when blood serum Ca concentration is < 6.0 mg/dL and subclinical hypocalcemia when serum total Ca concentration is < 8.0 mg/dL^83^. Plasma Ca concentration decreases significantly around the time of calving^83^. Dairy cows typically excrete 30 to 40 g Ca per day in early lactation by producing colostrum (with 1.7–2.3 g Ca per kg) or milk (with 1.2 g Ca per kg)^85^. The reduction in plasma Ca levels reduces Ca stores in smooth muscle, especially in the sarcoplasmic network of skeletal muscle. Thus, the absence of uterine contractions or uterine fatigue, as well as abdominal muscle contractions, may prolong the parturition process in cattle and lead to dystocia^86^ and stillbirth.

### Study limitations and possible applications

This study had the limitation of not being able to account for fetal presentations/abnormalities or distinguish between calves that died within 24 h and calves that were stillborn. The study was also limited by the fact that only one blood sample was collected at calving. Further studies with multiple blood samples around calving and to discover new biomarkers associated with dystocia and stillbirth would be helpful. Several attempts have been made to reduce the incidence of dystocia. For example, selection for low calf birth weight relative to expected sire progeny differences could contribute to the smaller skeletal size and lower calf birth weight, especially in heifers^44^. Also, selection for a larger pelvic area (pelvic height and width) as well as measuring internal pelvic dimensions and culling heifers with abnormally shaped or very small pelvic areas before breeding could help reduce the incidence of dystocia during the first parturition^87,88^. It is possible to reduce twinning as a risk factor of stillbirths in high-yielding dairy cows with a two-track approach. Double ovulation and dizygotic twins can be reduced by manipulating ovarian function to increase progesterone during growth of the preovulatory follicle before timed artificial insemination^89–91^. Second, cows diagnosed with bilateral twins early in gestation should be allowed to continue gestation with additional support at calving, whereas selective reduction may be attempted in cows diagnosed with unilateral twins^91^. Intensive monitoring of parturition is necessary to reduce the incidence of dystocia and stillbirth^92–94^. Several technologies are available to monitor the onset of calving^94–97^. These include inclinometers and accelerometers that detect tail raising and behavioural changes, intravaginal thermometers that detect allantochorion expulsion and body temperature drop, abdominal belts that monitor uterine contractions, and devices that monitor calf expulsion in the vaginal or vulvar lips^94,98,99^. To reduce the incidence of stillbirths, dairy cows can be monitored more frequently with a surveillance camera system before calving^100^. Evidence suggests that farmer obstetric skills and calving management training are associated with a reduction in stillbirth rates^101,102^. A previous study on an Irish dairy farm concluded that lack of monitoring of the second stage of parturition (expulsion of the calf) resulted in a higher incidence of dystocia in cattle^103^. This was recently confirmed by a Hungarian study in which monitoring the onset of calving with a calving alarm thermometer reduced the incidence of dystocia and improved postpartum health of the dam and newborn calf survival^95^.

## Conclusions

Based on our results, the most important risk factors identified by RF for dystocia were length of dry period, calf birth weight, and parity. Cows with a long dry period of more than 100 days were associated with the highest incidence of dystocia, while cows with a short dry period between 46 and 60 days had the lowest incidence. Our results showed that the incidence of dystocia in cows that delivered stillborn calves was higher in twins than in singletons. This study found that cows giving birth to twins have an increased risk of dystocia if the calves weigh more than 44 kg, suggesting that twin calves with a higher birth weight have a greater impact on feto-pelvic disproportion than singletons. The major risk factors identified by RF for stillbirths were twin status, parity, length of dry period, and calf birth weight. However, at the cow level, several risk factors interact to cause stillbirths in dairy cows. In cows that gave birth to twins, the risk of stillbirth was greater in cows with parity > 4 than in other parities. One finding of this study was that cows with a dry period > 100 days had a higher risk of stillbirth in cows that gave birth to twins. Compared to other seasons, a higher rate of stillbirths was observed in cows calving twins in summer. Finally, calving in summer may increase the risk of stillbirth if calves are born at low birth weight (≤ 35 kg). As indicated by the data, cows that were hypo-calcemic at the time of parturition had a higher risk of dystocia and stillbirth.

## Materials and methods

### Farms and data collection

Isfahan province is located in a geographical area of 32 380 4100 N, 51 400 300 E with 4 different seasons. The criteria for selection of herds were (1) accurate recording system and completeness of data used in the model for dystocia and stillbirths, (2) possibility of blood sampling, and (3) herd size (farms with > 2500 dairy cows). Only 3 of 8 dairy farms with approximately 2500 to 4800 dairy cows were included in the study. Calving seasons were defined as spring (March to May), summer (June to August), autumn (September to November), and winter (December to February). The average annual temperature is 18.5 °C and ranges from − 10 °C (in January) to 41 °C (in July) with an average annual precipitation of 122 mm. The relative humidity typically ranges from 10 to 88%, with an average of 30% during 2011–2017 (Iran Meteorological Organization, 2018). Ethical approval for all procedures involving animals was obtained from the Animal Care and Use Committee of Isfahan University of Technology (IACUC#2010/05) before the study began. The study complies with ARRIVE guidelines for reporting in vivo experiments and all methods were performed in accordance with the relevant guidelines and regulations. Informed consents were obtained from the owners of the animals used in the study for the purpose of research and publication.

Performance and pedigree were officially recorded on all three dairy farms. During the dry- period, all cows were housed in sand-bedded freestall barns. All cows were moved to prepartum pens 21 ± 5 days before the expected calving date and were closely monitored by trained personnel and/or veterinarians on the farm. Cows were checked every 3–5 h for signs of imminent parturition (i.e., udder enlargement, milk let-down, relaxation of the tail ligament). After calving, all cows were moved to fresh housing pens with sand-bedded freestall barns where they remained for approximately 30 ± 4 days. Subsequently, cows were relocated to mid-lactation pens and fed regular lactation feed for ad libitum intake according to the farm's standard procedure. Cows were milked three times a day at 0800, 1600, and 0000 h (± 1 h) in a milking parlour equipped with mechanical ventilation (fan and spray system). Cows were fed a balanced total mixed ration (TMR) consisting of approximately 40% forage (corn silage, alfalfa hay, and straw) and 60% concentrate (i.e., barley grain, corn grain, beet pulp, soybean meal, canola meal, cottonseed meal, meat meal, protected fat powder, and vitamin supplements, sodium bicarbonate, macro-minerals, microminerals, salt, and feed additives). Although each herd had its feeding and management policy, all three dairy farms had regular veterinary care, artificial insemination, heat synchronization, and vaccination. All dairy cows were artificially inseminated, usually with semen from North American and European sires. Holstein cows from these dairy farms were subjected to the Ovsynch protocol by consulting veterinarians.

All cows were milked at the first scheduled milking time (within 2 to 6 h) after calving. Calves from dairy herds were housed individually in straw-bedded pens indoor in temperature-controlled buildings and received 5–6 l of colostrum during the first 12 h of life (2.5–3 L by 1 h after birth and 2.5–3 L at 12 h after the first feeding). In the case of twin birth, the birth weights of both calves were considered separately. Data on Freemartin heifers were excluded from the analysis. Calves were then housed outdoors in individual pens with free access to water, hay, and starter feed until weaning when they were moved to group housing. Routine veterinary care of milk-fed calves was provided by herd veterinarians.

The study was conducted in two phases. Phase 1 examined cow-level risk factors influencing dystocia and stillbirth, while phase 2 examined the likely associations between selected blood macro-minerals and dystocia and stillbirth. All data were collected throughout the study by veterinarians on each farm using the same farm management software system. Cows were identified by their unique ID number. The original data set was edited to ensure reliability and consistency for statistical analysis. Therefore, records with missing or ambiguous calving dates, incorrect evaluation dates (for drying off, breeding, parturition), missing parity number, BW outside 15 to 70 kg, gestation length of less than 260 or more than 300 days, and an outlier in selected blood macro-minerals were excluded from the analysis. For the identification of outliers in selected blood macro-minerals, box plots of each raw data set were checked, and Z-standardization was performed. Outliers were identified as values with a standard deviation > 2. Phase 1 data consisted of 51,405 calving records from 14,546 Holstein cows collected between April 2011 and September 2017. In Phase 2, blood samples were collected from a random subset of those cows comprising 1311 animals along with other management information between April and September 2017. Randomization was performed at cow level by a computer-generated random number list when visiting each farm at least once a week on the same day for blood collection from all calving cows.

### Blood sampling and analysis

To investigate the association between selected macro-minerals in blood at the time of parturition (1 h before to 1 h after calving) and the incidence of dystocia and stillbirth, blood samples from 1311 cows were collected via the coccygeal vessels in evacuated tubes containing an anticoagulant (EDTA) at the time of parturition from April to September 2017. Samples were then centrifuged at 1300×*g* for 15 min and then stored at − 20 °C until analysis. Plasma concentrations of Ca, phosphorus (P), and Mg were measured with an autoanalyzer (Alcyon 300, Abbott Laboratories, Abbott Park, IL, USA) using a diagnostic kit (Pars Azmoon Co., Tehran, Iran) according to the manufacturer's instructions.

### Statistical analysis

Screening the dataset was conducted using the SQL Server software^104^ running on Microsoft Windows 10 (Pro × 64) operating system. Statistical analyses were conducted using SAS statistical software v.9.4 (SAS Institute, Inc. Cary, North Carolina; http://www.sas.com). Multivariable logistical regression procedures (PROC LOGISTIC) with backward elimination method through the maximum likelihood method of PROC GLIMMIX in SAS was performed to evaluate factors associated with the incidence of dystocia and stillbirth. At first, full data analysis including the main factors and all two-way interaction between variables was done. Interaction terms that did not significantly contribute to the regression model for dystocia and stillbirth were removed from the model one by one. Different models were compared using the Akaike information criteria (AIC) to evaluate the goodness-of-fit of the models. Also, multicollinearity among variables was evaluated in the model by computing the variance inflation factor (VIF). The homogeneity of variances (homoscedasticity) was verified by the specification of the COVTEST homogeneity command in SAS. An optimal model was selected based on the minimum AIC and the significance of the main effect or interaction associated with dystocia and stillbirth. The final models were as follows:

***Dystocia***

$$\begin{aligned} Logit(\pi ) & = \alpha + {\mathrm{Cyear}}_{i} {\mathrm{ + Cseason}}_{j} {\mathrm{ + Parity}}_{k} {\mathrm{ + Drylen}}_{l} {\mathrm{ + BCS}}_{m} {\mathrm{ + TWN}}_{n} {\mathrm{ + S}}{\mathrm{ex}}_{o} {\mathrm{ + Cweight}}_{p} {\mathrm{ + SB}}_{q} \\ & \quad { + }\beta_{1r} {\mathrm{(Preg}}_{ils} {) + }\beta_{2s} {\mathrm{(AFC}}_{ijk} {)} + {\mathrm{ Herd}}_{t} + SB_{k} \times {\mathrm{TWN}}_{n} + {\mathrm{TWN}}_{n} \times {\mathrm{Cweight}}_{q} \\ \end{aligned}$$

***Stillbirth***

$$\begin{aligned} Logit(\pi ) & = \alpha + {\mathrm{ Cyear}}_{i} {\mathrm{ + Cseason}}_{j} {\mathrm{ + Parity}}_{k} {\mathrm{ + TWN}}_{l} {\mathrm{ + S}}{\mathrm{ex}}_{m} {\mathrm{ + Cweight}}_{n} {\mathrm{ + Dys}}_{o} {\mathrm{ + BCS}}_{p} Drylen_{q} \\ & \quad + \beta_{1r} {\mathrm{(Preg}}_{ijk} {) + }\beta_{2s} {\mathrm{(AFC}}_{ijk} {\mathrm{) + Herd}}_{t} + Cseason_{j} \times TWN_{l} + \, Cseason_{j} \times {\mathrm{Cweight}}_{n} \\ & \quad + Parity_{k} \times TWN_{l} + Drylen_{q} \times TWN_{l} + {\mathrm{TWN}}_{l} \times {\mathrm{Cweight}}_{o} { + }Sex_{m} \times Cweight_{n} \\ \end{aligned}$$

where π = the odds probability of dystocia or stillbirth; α = intercept parameter; *Cyear* = fixed effect of calving year; *Cseason* = fixed effect of calving season; *Parity* = fixed effect of parity effect (l = 1, 2, 3, ≥ 4); *Drylen* = fixed effect of dry period length; *BCS* = fixed effect of body condition (BCS = 1, 2 and 3); *TWN* = fixed effect of twin status (1 = single birth, 2 = twin birth); *Sex* = fixed effect of calf sex (1 = male and 2 = female); *Cweigth* = fixed effect of calf weight at parturition (*Cweigth* = 1, 2, 3, …, 6); SB = fixed effect of calving status (0 = normal calving, 1 = stillbirth) *dys* = fixed effect of calving type (0 = easy calving, 1 = dystocia); β_1_ = regression coefficient of observations on gestation length (*Preg*) and age at first calving (*AFC*) as covariates and *Herd* = random effect. The association between dystocia and stillbirth incidence and each potential risk factor was investigated by odds ratio (OR) and its 95% CI. The incidence of dystocia or stillbirth was calculated by dividing the number of cases of dystocia or stillbirth per year by the total calving.

The statistical models employed for the selected blood macro-minerals were as follows:

$$\begin{aligned} Logit(\pi ) & = \alpha + Parity_{i} + Drylen_{j} + BCS_{k} + Sex_{l} + Cweight_{m} \\ & \quad + CaL_{n} + PL_{o} + MGl_{p} + \beta_{1q} (\Pr eg) + Herd_{r} \\ \end{aligned}$$

where π = the odds probability of dystocia or stillbirth; α = the intercept parameter; *Parity*_*i*_ = fixed effect of parity effect (l = 1, 2, 3, ≥ 4); *Drylen*_*j*_ = fixed effect of dry period length; *BCS*_*k*_ = the fixed effect of body condition (BCS = 1, 2 and 3); *Sex*_*l*_ = fixed effect of calf sex (1 = male and 2 = female); *Cweigth*_*m*_ = fixed effect of calf weight at parturition (*Cweigth* = 1, 2, 3, …, 6); $CaL_{n}$: fixed effect of Ca concentrations levels (1, and 2); $PL_{o}$: fixed effect of P concentrations levels (1, and 2); $MgL_{p}$: fixed effect of Mg concentrations levels (1, and 2); β_1_ = regression coefficient of observations on gestation length (*Preg*) as a covariate and *Herd*_*r*_ = random effect. Statistical significance was considered when *P* ≤ 0.05; a trend was considered when 0.05 < *P* ≤ 0.10.

Random forest was performed to rank cow-level risk factors based on their predictive value for dystocia and stillbirth from the machine learning module in JASP (Version 0.14)^105^. The dataset was split into three subsets: the training set (60%), the validation set (20%), and the test (20%). Classification performance is a report based on several metrics. For true positive (TP) as the number true positives, false positive (FP) as the number of false positives, true negative (TN) as the number of true negatives, and false negative (FN) as the number of false negatives, we measured the following:

$$\begin{aligned} {\mathrm{Sensitivity}} & = {\mathrm{TP}}/\left( {{\mathrm{TP}} + {\mathrm{FN}}} \right) \\ {\mathrm{Specificity}} & = {\mathrm{TN}}/\left( {{\mathrm{TN}} + {\mathrm{FP}}} \right) .\\ \end{aligned}$$

## Supplementary Information

Supplementary Information.

## Data Availability

The data that support the findings of this study are available from the corresponding author, M.H. Ghaffari, upon reasonable request.
