# Supplementary material for: Identification of cow-level risk factors and associations of selected blood macro-minerals at parturition with dystocia and stillbirth in Holstein dairy cows
Source: Sci Rep. 2022 Apr 8;12:5929. doi: 10.1038/s41598-022-09928-w (PMC8993806; doi:10.1038/s41598-022-09928-w)
Supplement: Supplementary file 1 — Supplementary Information. [file 41598_2022_9928_MOESM1_ESM.docx]

**Supplementary material**

**Identification of cow-level risk factors and associations of selected blood macro-minerals at parturition with dystocia and stillbirth in Holstein dairy cows**

**M. Bahrami-Yekdangi^1^, G. R. Ghorbani^2^, A. Sadeghi-Sefidmazgi^2^, A. Mahnani^2^, J. K. Drackley^3^, M. H. Ghaffari^4*^**

^1^ Animal Science Research Institute of Iran, Agricultural Research, Education and Extension Organization (AREEO), 3146618361 Karaj, Iran. ^2^Department of Animal Sciences, College of Agriculture, Isfahan University of Technology, PO Box 84156-83111, Isfahan, Iran. ^3^Department of Animal Sciences, University of Illinois, Urbana 61801, USA. ^4^ Institute of Animal Science, University of Bonn, 53111 Bonn, Germany

*Corresponding author: Email: morteza1@uni-bonn.de

**Author Contributions**

M.B. and G.R.G., M.H.G. supervised the study. M.B. conducted the farm trial. M.H.G. served as a scientific advisor. A. S., A. M., and M.H.G. did data analysis and visualization. M.H.G performed interpretation and wrote the original draft. M.H.G. revised the manuscript for intellectual content. J. K. D., and M. H. G. revised the manuscript. All authors read and approved the final manuscript.

**Supplemental Table S1A.**  Estimated odds ratios and 95% confidence levels (CI) of variables included in the binary logistic regression model of the generalized linear mixed model for cow-level risk factors for dystocia incidence in Holstein dairy cows (n = 51,405).

| Variable | Odd ratio |
| --- | --- |
|  | (95% CI) |
| Calving Year |  |
| 2011 | Referent |
| 2012 | 0.95 (0.85-1.05) |
| 2013 | 1.00 (0.90-1.11) |
| 2014 | 1.10 (1.00-1.22) |
| 2015 | 1.34 (1.22-1.48) |
| 2016 | 1.00 (0.91-1.11) |
| 2017 | 1.09 (0.97-1.23) |
| Calving season |  |
| Spring | Referent |
| Summer | 0.99 (0.92-1.06) |
| Autumn | 1.01 (0.94-1.09) |
| Winter | 1.08 (1.00-1.17) |
| Parity |  |
| 1 | Referent |
| 2 | 0.70 (0.64 -0.75) |
| 3 | 0.73 (0.66-0.80) |
| ≥ 4 | 0.86 (0.79-0.94) |
| Twinning status |  |
| Single birth | Referent |
| Twin birth | 1.52 (1.24-1.87) |
| Twin birth × Calf birth weight |  |
| Twin birth × Stillbirth |  |
| Dry period length |  |
| ≤ 45 | Referent |
| 46-60 | 0.47 (0.44-0.50) |
| 61-100 | 0.61 (0.56-0.67) |
| > 100 | 1.05 (0.94-1.18) |
| Gestation length |  |
| Calf birth weight |  |
| ≤ 35 | Referent |
| 35.1-38 | 0.88 (0.75-1.03) |
| 38.1-40 | 0.87 (0.72-1.05) |
| 40.1-42 | 0.80 (0.61-1.05) |
| 42.1-44 | 1.09 (0.83-1.42) |
| 44.1-46 | 0.91 (0.59-1.40) |
| 46 < | 1.77 (1.48-2.12) |
| Calf sex |  |
| Female | Referent |
| Male | 1.36 (1.29-1.43) |
| Stillbirth |  |
| No |  |
| Yes | 3.04 (2.54-3.63) |
| BCS |  |
| < 3.25 | Referent |
| 3.25 -3.75 | 1.09 (1.03-1.15) |
| > 3.75 | 1.27 (1.13-1.42) |

**Supplemental Table S1B:** Estimated odds ratios and 95% confidence levels (CI) of significant interactions between the cow-level risk factors associated with dystocia included in the binary logistic regression model of the generalized linear mixed model in Holstein dairy cows (n = 51,405).

| **Item** ^§^ | **OR (CI 95%)** | **P-value** |
| --- | --- | --- |
| **Twin status × Stillbirth** |  |  |
| Single with Stillbirth × Single without Stillbirth | 2.48 (1.049-3.485) | 0.012 |
| Twin without Stillbirth × Single without Stillbirth | 1.52 (0.79-0.94) | < 0.01 |
| Twin with Stillbirth × Single without Stillbirth | 4.65 (3.832 -5.644) | < 0.01 |
| Twin without Stillbirth × Single with Stillbirth | 0.503 (0.361-0.701) | < 0.01 |
| Twin with Stillbirth × Single with Stillbirth | 3.727 (2.882- 4.819) | < 0.01 |
| Twin with Stillbirth × Twin without Stillbirth | 7.415 (5.43- 10.126) | < 0.01 |
| **Twin birth × Calf birth weight** |  |  |
| Single - BW2 × Single - BW1 | 1.01 (0.88- 1.10) | 0.8683 |
| Single - BW3 × Single - BW1 | 1.16 (1.04 -1.30) | 0.008 |
| Single - BW4 × Single - BW1 | 1.24 (1.10-1.39) | < 0.01 |
| Single - BW5 × Single - BW1 | 1.51 (1.34-1.68) | < 0.01 |
| Single - BW6 × Single - BW1 | 1.88 (1.67- 2.12) | < 0.01 |
| Single - BW7 × Single - BW1 | 3.27 (2.93-3.64) | < 0.01 |
| Twin - BW1 × Single - BW1 | 3.11 (2.43-3.97) | < 0.01 |
| Twin - BW2 × Single - BW1 | 1.63 (0.97-2.71) | < 0.01 |
| Twin - BW3 × Single - BW1 | 1.38 (0.60 -3.18) | 0.4474 |
| Twin - BW4 × Single - BW1 | 2.46 (1.50-4.05) | 0.0629 |
| Twin - BW5 × Single - BW1 | 2.04 (1.45-2.88) | < 0.01 |
| Twin - BW6 × Single - BW1 | 2.45 (1.84-3.25) | < 0.01 |
| Twin - BW7 × Single - BW1 | 3.00 (2.16-4.17) | < 0.01 |
| Single - BW3 × Single - BW2 | 1.17 (1.06-1.30) | < 0.01 |
| Single - BW4 × Single - BW2 | 1.25 (1.28-1.39) | < 0.01 |
| Single - BW5 × Single - BW2 | 1.52 (1.37-1.69) | < 0.01 |
| Single - BW6 × Single - BW2 | 1.90 (1.70-2.12) | < 0.01 |
| Single - BW7 × Single - BW2 | 3.30 (2.99-3.63) | < 0.01 |
| Twin - BW1 × Single - BW2 | 1.64 (0.98-2.73) | 0.0572 |
| Twin - BW2 × Single - BW2 | 1.39 (0.61-3.21) | 0.4337 |
| Twin - BW3 × Single - BW2 | 2.06 (1.46-2.90) | < 0.01 |
| Twin - BW4 × Single - BW2 | 3.14 (2.46-3.99) | < 0.01 |
| Twin - BW5 × Single - BW2 | 2.48 (1.51-4.08 | < 0.01 |
| Twin - BW6 × Single - BW2 | 2.47 (1.87-3.27) | < 0.01 |
| Twin - BW7 × Single - BW2 | 3.03 (2.19-4.20) | < 0.01 |
| Single - BW4 × Single - BW3 | 1.07 (0.96-1.18) | 0.21 |
| Single - BW5 × Single - BW3 | 1.28 (1.17-1.44) | < 0.01 |
| Single - BW6 × Single - BW3 | 1.62 (1.45-1.80) | < 0.01 |
| Single - BW7 × Single - BW3 | 2.81 (2.56-3.09) | < 0.01 |
| Twin - BW1 × Single - BW3 | 1.75 (1.2-2.47) | < 0.01 |
| Twin - BW2 × Single - BW3 | 1.40 (0.84-2.33) | 0.1957 |
| Twin - BW3 × Single - BW3 | 2.68 (2.10-3.41) | < 0.01 |
| Twin - BW4 × Single - BW3 | 2.11 (1.59-2.79) | < 0.01 |
| Twin - BW5 × Single - BW3 | 1.19 (0.52-2.74) | 0.6821 |
| Twin - BW6 × Single - BW3 | 2.12 (1.29-3.48) | < 0.01 |
| Twin - BW7 × Single - BW3 | 2.58 (1.87-3.58) | < 0.01 |
| Single - BW5 × Single - BW4 | 1.21 (1.09-1.35) | < 0.01 |
| Single - BW6 × Single - BW4 | 1.52 (1.36-1.69) | < 0.01 |
| Single - BW7 × Single - BW4 | 2.63 (2.40-2.89) | < 0.01 |
| Twin - BW1 × Single - BW4 | 1.11 (0.48-2.56) | 0.7998 |
| Twin - BW2 × Single - BW4 | 1.97 (1.49-2.62) | < 0.01 |
| Twin - BW3 × Single - BW4 | 1.65 (1.17-2.32) | < 0.01 |
| Twin - BW4 × Single - BW4 | 1.31 (0.79-2.18) | 0.2991 |
| Twin - BW5 × Single - BW4 | 1.98 (1.21-3.26) | 0.0067 |
| Twin - BW6 × Single - BW4 | 2.50 (1.96-3.19) | < 0.01 |
| Twin - BW7 × Single - BW4 | 2.42 (1.74-3.35) | < 0.01 |
| Single - BW6 × Single - BW5 | 1.25 (1.12-1.39) | < 0.01 |
| Single - BW7 × Single - BW5 | 2.17 (1.98-2.37) | < 0.01 |
| Twin - BW1 × Single - BW5 | 2.06 (1.62-2.63) | < 0.01 |
| Twin - BW2 × Single - BW5 | 1.63 (1.23-2.15) | < 0.01 |
| Twin - BW3 × Single - BW5 | 1.35 (0.96-1.90) | 0.0802 |
| Twin - BW4 × Single - BW5 | 1.08 (0.65-1.80) | 0.7683 |
| Twin - BW5 × Single - BW5 | 1.63 (1.00-2.68) | 0.0515 |
| Twin - BW6 × Single - BW5 | 1.92 (1.40-2.11) | 0.8391 |
| Twin - BW7 × Single - BW5 | 1.99 (1.44-2.77) | < 0.01 |
| Single - BW7 × Single - BW6 | 1.74 (1.58-1.91) | < 0.01 |
| Twin - BW1 × Single - BW6 | 1.65 (1.29-2.11) | < 0.01 |
| Twin - BW2 × Single - BW6 | 1.30 (0.98-1.73) | 0.0676 |
| Twin - BW3 × Single - BW6 | 1.09 (0.77-1.53) | 0.6378 |
| Twin - BW4 × Single - BW6 | 0.86 (0.52-1.44) | 0.5776 |
| Twin - BW5 × Single - BW6 | 1.31 (0.80-2.15) | 0.287 |
| Twin - BW6 × Single - BW6 | 0.73 (0.32-1.69) | 0.4684 |
| Twin - BW7 × Single - BW6 | 1.6 (1.15-2.21) | 0.0051 |
| Twin - BW1 × Single - BW7 | 0.95 (0.75-1.21) | 0.6793 |
| Twin - BW2 × Single - BW7 | 1.75 (1.57-2.99) | 0.0419 |
| Twin - BW3 × Single - BW7 | 1.62 (1.45-2.88) | 0.0064 |
| Twin - BW4 × Single - BW7 | 1.5 (1.30-2.83) | 0.0071 |
| Twin - BW5 × Single - BW7 | 1.75 (0.46-2.23) | 0.2587 |
| Twin - BW6 × Single - BW7 | 1.42 (1.18-2.97) | 0.0423 |
| Twin - BW7 × Single - BW7 | 1.92 (1.66-2.27) | 0.6066 |
| Twin - BW2 × Twin- BW1 | 1.79 (1.59-2.05) | 0.1095 |
| Twin- BW3 × Twin- BW1 | 1.66 (1.46-2.94) | 0.0227 |
| Twin- BW4 × Twin- BW1 | 1.52 (1.30-2.89) | 0.0171 |
| Twin- BW5 × Twin- BW1 | 1.79 (1.47-2.34) | 0.3833 |
| Twin- BW6 × Twin- BW1 | 1.45 (1.19-2.04 | 0.0615 |
| Twin- BW7 × Twin- BW1 | 1.96 (0.69-1.35 | 0.8421 |
| Twin- BW3 × Twin- BW2 | 1.09 (0.56-1.23) | 0.3629 |
| Twin- BW4 × Twin- BW2 | 1.16 (0.88-1.35) | 0.1463 |
| Twin- BW5 × Twin- BW2 | 1.00 (0.58-1.73) | 0.9859 |
| Twin- BW6 × Twin- BW2 | 1.56 (1.28-2.33) | 0.1928 |
| Twin- BW7 × Twin- BW2 | 1.22 (0.84-1.78) | 0.2829 |
| Twin- BW4 × Twin- BW3 | 1.28 (0.84-1.43) | 0.4478 |
| Twin- BW5 × Twin- BW3 | 1.20 (0.68-2.18) | 0.5258 |
| Twin- BW6 × Twin- BW3 | 1.68 (1.28-2.64) | 0.3864 |
| Twin- BW7 × Twin- BW3 | 1.47 (0.96-2.25) | 0.0762 |
| Twin- BW5 × Twin- BW4 | 1.51 (0.76-3.02) | 0.2404 |
| Twin- BW6 × Twin- BW4 | 1.35 (0.92-2.22) | 0.7401 |
| Twin- BW7 × Twin- BW4 | 1.85 (1.04-3.288) | 0.0373 |
| Twin- BW6 × Twin- BW5 | 1.78 (1.22-2.46) | 0.236 |
| Twin- BW7 × Twin- BW5 | 1.22 (0.69-2.15) | 0.4926 |
| Twin- BW7 × Twin- BW6 | 2.17 (0.90-5.22) | 0.0829 |

§ BW= Calf birth weight

BW1-7: 1 = ≤ 35 kg, 2 = 35.1-38 kg, 3 = 38.1- 40 kg, 4 = 40.1 -42 kg, 5 = 42.1-44 kg, 6 = 44.1- 46 kg, 7 = > 46 kg.

**Supplemental Table S2:** Estimated odds ratios and 95% confidence levels (CI) of selected blood macro-minerals at parturition included in the binary logistic regression model of the generalized linear mixed model for dystocia incidence in Holstein dairy cows (n = 1,311)

| **Variable** | **Odds ratio (95% CI)** |
| --- | --- |
| Calcium (mg/dl) |  |
| Hypo (≤ 8) | Referent |
| Normal (> 8.1) | 0.45 (0.23-0.91) |
| Phosphorus (mg/dl) |  |
| Hypo (≤ 4) | Referent |
| Normal (> 4.1) | 0.82 (0.71-1.03) |
| Magnesium (mg/dl) |  |
| Hypo (≤ 2.5) | Referent |
| Normal (> 2.51) | 1.03 (0.92-1.19) |

**Supplemental Table S3A:** Estimated odds ratios and 95% confidence levels (CI) of variables included in the binary logistic regression model of the generalized linear mixed model for cow-level risk factors for stillbirth incidence in Holstein dairy cows (n = 51,405).

| Variable | Odds ratio (95% CI) |
| --- | --- |
| Calving Year |  |
| 2011 | Referent |
| 2012 | 1.02 (0.79-1.19) |
| 2013 | 1.05 (0.86-1.29) |
| 2014 | 0.79 (0.64-0.97) |
| 2015 | 0.70 (0.57-0.86) |
| 2016 | 0.37 (0.29-0.46) |
| 2017 | 0.29 (0.22-0.39) |
| Calving season |  |
| Spring | Referent |
| Summer | 1.05 (0.86-1.29) |
| Autumn | 0.78 (0.63-0.95) |
| Winter | 0.77 (0.62- 0.96) |
| Season × Twinning status |  |
| Season × Calf birth weight |  |
| Parity |  |
| 1 | Referent |
| 2 | 1.98 (1.65 -2.36) |
| 3 | 1.84 (1.48- 2.29) |
| 4 ≤ | 2.20 (1.69- 2.86) |
| Parity × Twinning status |  |
| Twinning status |  |
| Single birth | Referent |
| Twin birth | 3.39 (2.85-3.55) |
| Dry period length |  |
| < 45 d | Referent |
| 46-60 d | 1.21 (1.03-1.41) |
| 61 -100 d | 1.30 (1.03-1.66) |
| >100 d | 1.25(0.88-1.78) |
| Dry period length × Twinning status |  |
| Gestation length | 0.99 |
| Calf birth weight |  |
| ≤ 35 | Referent |
| 35.1-38 | 0.64 (0.51-0.80) |
| 38.1-40 | 0.43 (0.34-0.54) |
| 40.1-42 | 0.33 (0.25-0.43) |
| 42.1-44 | 0.31 (0.23 -0.41) |
| 44.1-46 | 0.24 (0.16 -0.36) |
| 46 < | 0.54 (0.42-0.70) |
| Calf sex |  |
| Female | Referent |
| Male | 1.16 (1.04-1.29) |
| Dystocia |  |
| No | Referent |
| Yes | 2.04 (1.78-2.34) |

**Supplemental Table S3B:** Calving number for each item was included in the significant interaction between cow-level risk factors associated with dystocia in the binary logistic regression model of the generalized linear mixed model in Holstein dairy cows (n = 51,405).

| **Item** ^§^ | **Calving No.** |
| --- | --- |
| Single without Stillbirth | 48594 |
| Single with Stillbirth | 997 |
| Twin without Stillbirth | 597 |
| Twin with Stillbirth | 1217 |
| Single - BW1 | 6191 |
| Single - BW2 | 8043 |
| Single - BW3 | 8077 |
| Single - BW4 | 6957 |
| Single - BW5 | 6963 |
| Single - BW6 | 4899 |
| Single - BW7 | 8461 |
| Twin - BW1 | 615 |
| Twin - BW2 | 381 |
| Twin - BW3 | 258 |
| Twin - BW4 | 135 |
| Twin - BW5 | 122 |
| Twin - BW6 | 61 |
| Twin - BW7 | 242 |

§ BW = Calf birth weight

BW1-7: 1 = ≤ 35 kg, 2 = 35.1-38 kg, 3 = 38.1- 40 kg, 4 = 40.1 -42 kg, 5 = 42.1-44 kg, 6 = 44.1- 46 kg, 7 = > 46 kg.

**Supplemental Table S3C:** Estimated odds ratios (OR) and 95% confidence intervals (CI) for significant interactions among cow-level risk factors associated with stillbirths included in the binary logistic regression model of the generalized linear mixed model in Holstein dairy cows (n = 51,405).

| **Item^§^** | **OR** | **CI 95%** | | **P-value** |
| --- | --- | --- | --- | --- |
| **Calving season× Twin birth** |  |  |  |  |
| Spring - TWN vs. Spring - ST | 206.95 | 147.90 | 289.58 | <.0001 |
| Summer - ST vs. Spring - ST | 0.92 | 0.75 | 1.11 | 0.3656 |
| Summer - TWN vs. Spring - ST | 259.46 | 188.90 | 356.38 | <.0001 |
| Autumn - ST vs. Spring - ST | 1.09 | 0.90 | 1.31 | 0.3978 |
| Autumn - TWN vs. Spring - ST | 135.84 | 98.76 | 186.84 | <.0001 |
| Winter - ST vs. Spring - ST | 1.09 | 0.79 | 1.20 | 0.8103 |
| Winter - TWN vs. Spring - ST | 123.01 | 87.76 | 172.41 | <.0001 |
| Summer - ST vs. Spring - TWN | 0.01 | 0.00 | 0.01 | <.0001 |
| Summer - TWN vs. Spring - TWN | 1.25 | 0.90 | 1.74 | 0.179 |
| Autumn - ST vs. Summer - TWN | 0.01 | 0.00 | 0.01 | <.0001 |
| Autumn - TWN vs. Summer - TWN | 0.66 | 0.47 | 0.92 | 0.0156 |
| Winter - ST vs. Summer - TWIN | 0.01 | 0.00 | 0.01 | <.0001 |
| Winter - TWIN vs. Summer - TWN | 0.59 | 0.42 | 0.85 | 0.0041 |
| Summer - TWN vs. Summer - ST | 283.72 | 209.97 | 383.37 | <.0001 |
| Autumn - ST vs. Summer - ST | 1.19 | 1.00 | 1.41 | 0.0536 |
| Autumn - TWN vs. Summer - ST | 148.54 | 108.99 | 202.45 | <.0001 |
| Winter -ST vs. Summer - ST | 1.07 | 0.88 | 1.29 | 0.5091 |
| Winter -TWN vs. Summer - ST | 134.51 | 96.77 | 186.97 | <.0001 |
| Summer - TWN vs. Autumn - ST | 0.00 | 0.00 | 0.01 | <.0001 |
| Summer - TWN vs. Autumn - TWN | 0.52 | 0.38 | 0.72 | <.0001 |
| Summer - TWN vs. Winter - ST | 0.00 | 0.00 | 0.01 | <.0001 |
| Summer - TWN vs. Winter - TWN | 0.47 | 0.34 | 0.66 | <.0001 |
| Autumn - TWN vs. Autumn - ST | 125.14 | 92.25 | 169.75 | <.0001 |
| Winter - ST vs. Autumn - ST | 0.90 | 0.75 | 1.08 | 0.2509 |
| Winter - TWN vs. Autumn - ST | 113.31 | 81.73 | 157.11 | <.0001 |
| Winter - ST vs. Autumn - TWN | 0.01 | 0.01 | 0.01 | <.0001 |
| Winter - TWN vs. Autumn - TWN | 0.91 | 0.65 | 1.26 | 0.5593 |
| Winter - TWN vs. Winter - ST | 126.14 | 90.34 | 176.12 | <.0001 |
| **Calving season× Calf birth weight** |  |  |  |  |
| Spring - BW_2_ vs. Spring - BW_1_ | 0.60 | 0.41 | 0.90 | 0.0123 |
| Spring - BW_3_ vs. Spring - BW_1_ | 0.56 | 0.37 | 0.84 | 0.0052 |
| Spring - BW_4_ vs. Spring - BW_1_ | 0.34 | 0.21 | 0.55 | <.0001 |
| Spring - BW_5_ vs. Spring - BW_1_ | 0.74 | 0.49 | 1.13 | 0.1634 |
| Spring - BW_6_ vs. Spring - BW_1_ | 0.43 | 0.25 | 0.74 | 0.0025 |
| Spring - BW_7_ vs. Spring - BW_1_ | 0.42 | 0.27 | 0.65 | 0.0001 |
| Summer - BW_1_ vs. Spring - BW_1_ | 1.07 | 0.76 | 1.52 | 0.6937 |
| Summer - BW_2_ vs. Spring - BW_1_ | 0.71 | 0.50 | 1.03 | 0.071 |
| Summer - BW_3_ vs. Spring - BW_1_ | 0.44 | 0.29 | 0.65 | <.0001 |
| Summer - BW_4_ vs. Spring - BW_1_ | 0.63 | 0.42 | 0.96 | 0.0301 |
| Summer - BW_5_ vs. Spring - BW_1_ | 0.42 | 0.27 | 0.65 | 0.0001 |
| Summer - BW_6_ vs. Spring - BW_1_ | 0.42 | 0.25 | 0.72 | 0.0014 |
| Summer - BW_7_ vs. Spring - BW_1_ | 0.67 | 0.46 | 0.99 | 0.0458 |
| Autumn - BW1 vs. Spring - BW1 | 0.74 | 0.51 | 1.06 | 0.1015 |
| Autumn - BW2 vs. Spring - BW1 | 0.47 | 0.32 | 0.69 | 0.0001 |
| Autumn - BW3 vs. Spring - BW1 | 0.37 | 0.25 | 0.56 | <.0001 |
| Autumn - BW4 vs. Spring - BW1 | 0.35 | 0.23 | 0.55 | <.0001 |
| Autumn - BW5 vs. Spring - BW1 | 0.42 | 0.28 | 0.65 | <.0001 |
| Autumn - BW6 vs. Spring - BW1 | 0.64 | 0.42 | 0.98 | 0.0414 |
| Autumn - BW7 vs. Spring - BW1 | 0.38 | 0.26 | 0.57 | <.0001 |
| Winter - BW_1_ vs. Spring - BW_1_ | 0.55 | 0.36 | 0.85 | 0.0064 |
| Winter - BW_2_ vs. Spring - BW_1_ | 0.55 | 0.36 | 0.83 | 0.004 |
| Winter - BW_3_ vs. Spring - BW_1_ | 0.44 | 0.29 | 0.68 | 0.0002 |
| Winter - BW_4_ vs. Spring - BW_1_ | 0.34 | 0.21 | 0.54 | <.0001 |
| Winter - BW_5_ vs. Spring - BW_1_ | 0.36 | 0.23 | 0.57 | <.0001 |
| Winter - BW_6_ vs. Spring - BW_1_ | 0.39 | 0.24 | 0.62 | <.0001 |
| Winter - BW_7_ vs. Spring - BW_1_ | 0.36 | 0.24 | 0.54 | <.0001 |
| Spring - BW_3_ vs. Spring - BW_2_ | 0.92 | 0.60 | 1.40 | 0.6917 |
| Spring - BW_4_ vs. Spring - BW_2_ | 0.56 | 0.35 | 0.91 | 0.0187 |
| Spring - BW_5_ vs. Spring - BW_2_ | 1.23 | 0.80 | 1.88 | 0.3525 |
| Spring - BW_6_ vs. Spring - BW_2_ | 0.71 | 0.41 | 1.24 | 0.2304 |
| Spring - BW_7_ vs. Spring - BW_2_ | 0.70 | 0.45 | 1.09 | 0.1142 |
| Summer - BW_1_ vs. Spring - BW_2_ | 1.78 | 1.24 | 2.55 | 0.0019 |
| Summer - BW_2_ vs. Spring - BW_2_ | 1.18 | 0.81 | 1.72 | 0.3851 |
| Summer - BW_3_ vs. Spring - BW_2_ | 0.72 | 0.48 | 1.08 | 0.1152 |
| Summer - BW_4_ vs. Spring - BW_2_ | 1.04 | 0.68 | 1.59 | 0.8453 |
| Summer - BW_5_ vs. Spring - BW_2_ | 0.69 | 0.44 | 1.08 | 0.1039 |
| Summer - BW_6_ vs. Spring - BW_2_ | 0.70 | 0.41 | 1.19 | 0.1852 |
| Summer - BW_7_ vs. Spring - BW_2_ | 1.11 | 0.75 | 1.65 | 0.5968 |
| Autumn - BW1 vs. Spring - BW2 | 1.22 | 0.84 | 1.78 | 0.2992 |
| Autumn - BW2 vs. Spring - BW2 | 0.78 | 0.52 | 1.15 | 0.2087 |
| Autumn - BW3 vs. Spring - BW2 | 0.62 | 0.41 | 0.93 | 0.0211 |
| Autumn - BW4 vs. Spring - BW2 | 0.58 | 0.37 | 0.91 | 0.0177 |
| Autumn - BW5 vs. Spring - BW2 | 0.70 | 0.46 | 1.08 | 0.1037 |
| Autumn - BW6 vs. Spring - BW2 | 1.06 | 0.69 | 1.63 | 0.7939 |
| Autumn - BW7 vs. Spring - BW2 | 0.63 | 0.42 | 0.94 | 0.0246 |
| Winter - BW_1_ vs. Spring - BW_2_ | 0.92 | 0.59 | 1.41 | 0.6904 |
| Winter - BW_2_ vs. Spring - BW_2_ | 0.91 | 0.60 | 1.38 | 0.6482 |
| Winter - BW_3_ vs. Spring - BW_2_ | 0.73 | 0.47 | 1.13 | 0.1554 |
| Winter - BW_4_ vs. Spring - BW_2_ | 0.56 | 0.35 | 0.90 | 0.017 |
| Winter - BW_5_ vs. Spring - BW_2_ | 0.60 | 0.38 | 0.95 | 0.0288 |
| Winter - BW_6_ vs. Spring - BW_2_ | 0.64 | 0.40 | 1.04 | 0.0704 |
| Winter - BW_7_ vs. Spring - BW_2_ | 0.59 | 0.39 | 0.90 | 0.0135 |
| Spring - BW_4_ vs. Spring - BW_3_ | 0.61 | 0.37 | 1.00 | 0.0508 |
| Spring - BW_5_ vs. Spring - BW_3_ | 1.34 | 0.86 | 2.07 | 0.1981 |
| Spring - BW_6_ vs. Spring - BW_3_ | 0.78 | 0.44 | 1.36 | 0.3765 |
| Spring - BW_7_ vs. Spring - BW_3_ | 0.76 | 0.49 | 1.20 | 0.2404 |
| Summer - BW_1_ vs. Spring - BW_3_ | 1.93 | 1.32 | 2.84 | 0.0008 |
| Summer - BW_2_ vs. Spring - BW_3_ | 1.29 | 0.87 | 1.91 | 0.2121 |
| Summer - BW_3_ vs. Spring - BW_3_ | 0.79 | 0.52 | 1.20 | 0.2647 |
| Summer - BW_4_ vs. Spring - BW_3_ | 1.14 | 0.73 | 1.76 | 0.57 |
| Summer - BW_5_ vs. Spring - BW_3_ | 0.75 | 0.47 | 1.19 | 0.2251 |
| Summer - BW_6_ vs. Spring - BW_3_ | 0.76 | 0.44 | 1.31 | 0.3209 |
| Summer - BW_7_ vs. Spring - BW_3_ | 1.21 | 0.80 | 1.83 | 0.3601 |
| Autmn - BW_1_ vs. Spring - BW_3_ | 1.33 | 0.90 | 1.97 | 0.158 |
| Autmn - BW_2_ vs. Spring - BW_3_ | 0.85 | 0.56 | 1.28 | 0.4245 |
| Autmn - BW_3_ vs. Spring - BW_3_ | 0.67 | 0.44 | 1.03 | 0.068 |
| Autmn - BW_4_ vs. Spring - BW_3_ | 0.63 | 0.40 | 1.01 | 0.0526 |
| Autmn - BW_5_ vs. Spring - BW_3_ | 0.76 | 0.49 | 1.19 | 0.2311 |
| Autmn - BW_6_ vs. Spring - BW_3_ | 1.15 | 0.74 | 1.80 | 0.5302 |
| Autmn - BW_7_ vs. Spring - BW_3_ | 0.69 | 0.46 | 1.04 | 0.0773 |
| Winter - BW_1_ vs. Spring - BW_3_ | 1.00 | 0.64 | 1.57 | 0.9893 |
| Winter - BW_2_ vs. Spring - BW_3_ | 0.99 | 0.64 | 1.53 | 0.9573 |
| Winter - BW_3_ vs. Spring - BW_3_ | 0.79 | 0.51 | 1.25 | 0.3162 |
| Winter - BW_4_ vs. Spring - BW_3_ | 0.61 | 0.38 | 1.00 | 0.0479 |
| Winter - BW_5_ vs. Spring - BW_3_ | 0.65 | 0.41 | 1.05 | 0.0769 |
| Winter - BW_6_ vs. Spring - BW_3_ | 0.70 | 0.43 | 1.14 | 0.154 |
| Winter - BW_7_ vs. Spring - BW_3_ | 0.65 | 0.42 | 0.99 | 0.0454 |
| Spring - BW_5_ vs. Spring - BW_4_ | 2.18 | 1.33 | 3.59 | 0.0021 |
| Spring - BW_6_ vs. Spring - BW_4_ | 1.27 | 0.69 | 2.33 | 0.4382 |
| Spring - BW_7_ vs. Spring - BW_4_ | 1.25 | 0.75 | 2.07 | 0.3929 |
| Summer - BW_1_ vs. Spring - BW_4_ | 3.16 | 2.02 | 4.95 | <.0001 |
| Summer - BW_2_ vs. Spring - BW_4_ | 2.10 | 1.33 | 3.32 | 0.0014 |
| Summer - BW_3_ vs. Spring - BW_4_ | 1.29 | 0.80 | 2.08 | 0.3047 |
| Summer - BW_4_ vs. Spring - BW_4_ | 1.86 | 1.13 | 3.05 | 0.0142 |
| Summer - BW_5_ vs. Spring - BW_4_ | 1.23 | 0.74 | 2.06 | 0.4287 |
| Summer - BW_6_ vs. Spring - BW_4_ | 1.24 | 0.68 | 2.24 | 0.4814 |
| Summer - BW_7_ vs. Spring - BW_4_ | 1.98 | 1.24 | 3.17 | 0.0042 |
| Autumn - BW1 vs. Spring - BW4 | 2.17 | 1.38 | 3.43 | 0.0009 |
| Autumn - BW2 vs. Spring - BW4 | 1.38 | 0.86 | 2.22 | 0.178 |
| Autumn - BW3 vs. Spring - BW4 | 1.10 | 0.68 | 1.78 | 0.6927 |
| Autumn - BW4 vs. Spring - BW4 | 1.04 | 0.62 | 1.73 | 0.8942 |
| Autumn - BW5 vs. Spring - BW4 | 1.25 | 0.76 | 2.05 | 0.3859 |
| Autumn - BW6 vs. Spring - BW4 | 1.89 | 1.14 | 3.11 | 0.0129 |
| Autumn - BW7 vs. Spring - BW4 | 1.13 | 0.70 | 1.81 | 0.6232 |
| Winter - BW_1_ vs. Spring - BW_4_ | 1.63 | 0.98 | 2.71 | 0.0587 |
| Winter - BW_2_ vs. Spring - BW_4_ | 1.62 | 0.99 | 2.64 | 0.0552 |
| Winter - BW_3_ vs. Spring - BW_4_ | 1.30 | 0.78 | 2.15 | 0.3106 |
| Winter - BW_4_ vs. Spring - BW_4_ | 1.00 | 0.58 | 1.71 | 0.9985 |
| Winter - BW_5_ vs. Spring - BW_4_ | 1.07 | 0.63 | 1.80 | 0.804 |
| Winter - BW_6_ vs. Spring - BW_4_ | 1.14 | 0.66 | 1.97 | 0.632 |
| Winter - BW_7_ vs. Spring - BW_4_ | 1.06 | 0.65 | 1.71 | 0.8287 |
| Spring - BW_6_ vs. Spring - BW_5_ | 0.58 | 0.33 | 1.02 | 0.0563 |
| Spring - BW_7_ vs. Spring - BW_5_ | 0.57 | 0.36 | 0.90 | 0.0148 |
| Summer - BW_1_ vs. Spring - BW_5_ | 1.45 | 0.97 | 2.16 | 0.0686 |
| Summer - BW_2_ vs. Spring - BW_5_ | 0.96 | 0.64 | 1.45 | 0.8582 |
| Summer - BW_3_ vs. Spring - BW_5_ | 0.59 | 0.38 | 0.91 | 0.0165 |
| Summer - BW_4_ vs. Spring - BW_5_ | 0.85 | 0.54 | 1.33 | 0.4804 |
| Summer - BW_5_ vs. Spring - BW_5_ | 0.56 | 0.35 | 0.90 | 0.0161 |
| Summer - BW_6_ vs. Spring - BW_5_ | 0.57 | 0.33 | 0.99 | 0.0452 |
| Summer - BW_7_ vs. Spring - BW_5_ | 0.91 | 0.60 | 1.38 | 0.6488 |
| Autumn - BW1 vs. Spring - BW5 | 1.00 | 0.66 | 1.49 | 0.9805 |
| Autumn - BW2 vs. Spring - BW5 | 0.63 | 0.42 | 0.97 | 0.0343 |
| Autumn - BW3 vs. Spring - BW5 | 0.51 | 0.33 | 0.78 | 0.002 |
| Autumn - BW4 vs. Spring - BW5 | 0.47 | 0.30 | 0.76 | 0.0019 |
| Autumn - BW5 vs. Spring - BW5 | 0.57 | 0.36 | 0.90 | 0.015 |
| Autumn - BW6 vs. Spring - BW5 | 0.86 | 0.55 | 1.36 | 0.527 |
| Autumn - BW7 vs. Spring - BW5 | 0.52 | 0.34 | 0.79 | 0.002 |
| Winter - BW_1_ vs. Spring - BW_5_ | 0.75 | 0.47 | 1.19 | 0.2162 |
| Winter - BW_2_ vs. Spring - BW_5_ | 0.74 | 0.47 | 1.16 | 0.1852 |
| Winter - BW_3_ vs. Spring - BW_5_ | 0.60 | 0.38 | 0.94 | 0.0268 |
| Winter - BW_4_ vs. Spring - BW_5_ | 0.46 | 0.28 | 0.75 | 0.002 |
| Winter - BW_5_ vs. Spring - BW_5_ | 0.49 | 0.30 | 0.79 | 0.0034 |
| Winter - BW_6_ vs. Spring - BW_5_ | 0.52 | 0.32 | 0.86 | 0.0111 |
| Winter - BW_7_ vs. Spring - BW_5_ | 0.48 | 0.31 | 0.75 | 0.001 |
| Spring - BW_7_ vs. Spring - BW_6_ | 0.98 | 0.56 | 1.73 | 0.9487 |
| Summer - BW_1_ vs. Spring - BW_6_ | 2.49 | 1.47 | 4.22 | 0.0007 |
| Summer - BW_2_ vs. Spring - BW_6_ | 1.66 | 0.97 | 2.83 | 0.0655 |
| Summer - BW_3_ vs. Spring - BW_6_ | 1.01 | 0.58 | 1.76 | 0.9665 |
| Summer - BW_4_ vs. Spring - BW_6_ | 1.46 | 0.83 | 2.58 | 0.1894 |
| Summer - BW_5_ vs. Spring - BW_6_ | 0.97 | 0.54 | 1.73 | 0.9126 |
| Summer - BW_6_ vs. Spring - BW_6_ | 0.97 | 0.51 | 1.88 | 0.9376 |
| Summer - BW_7_ vs. Spring - BW_6_ | 1.56 | 0.91 | 2.68 | 0.1081 |
| Autmn - BW_1_ vs. Spring - BW_6_ | 1.71 | 1.00 | 2.92 | 0.0496 |
| Autmn - BW_2_ vs. Spring - BW_6_ | 1.09 | 0.63 | 1.88 | 0.7628 |
| Autmn - BW_3_ vs. Spring - BW_6_ | 0.87 | 0.50 | 1.51 | 0.615 |
| Autmn - BW_4_ vs. Spring - BW_6_ | 0.82 | 0.45 | 1.46 | 0.4924 |
| Autmn - BW_5_ vs. Spring - BW_6_ | 0.98 | 0.56 | 1.73 | 0.9478 |
| Autmn - BW_6_ vs. Spring - BW_6_ | 1.49 | 0.84 | 2.62 | 0.1736 |
| Autmn - BW_7_ vs. Spring - BW_6_ | 0.89 | 0.51 | 1.53 | 0.662 |
| Winter - BW_1_ vs. Spring - BW_6_ | 1.28 | 0.72 | 2.29 | 0.399 |
| Winter - BW_2_ vs. Spring - BW_6_ | 1.27 | 0.72 | 2.24 | 0.4041 |
| Winter - BW_3_ vs. Spring - BW_6_ | 1.02 | 0.57 | 1.82 | 0.9413 |
| Winter - BW_4_ vs. Spring - BW_6_ | 0.79 | 0.43 | 1.44 | 0.4381 |
| Winter - BW_5_ vs. Spring - BW_6_ | 0.84 | 0.47 | 1.52 | 0.5654 |
| Winter - BW_6_ vs. Spring - BW_6_ | 0.90 | 0.49 | 1.65 | 0.7303 |
| Winter - BW_7_ vs. Spring - BW_6_ | 0.83 | 0.48 | 1.45 | 0.5112 |
| Summer - BW_1_ vs. Spring - BW_7_ | 2.54 | 1.68 | 3.82 | <.0001 |
| Summer - BW_2_ vs. Spring - BW_7_ | 1.69 | 1.11 | 2.57 | 0.0146 |
| Summer - BW_3_ vs. Spring - BW_7_ | 1.03 | 0.66 | 1.61 | 0.8927 |
| Summer - BW_4_ vs. Spring - BW_7_ | 1.49 | 0.94 | 2.35 | 0.0878 |
| Summer - BW_5_ vs. Spring - BW_7_ | 0.99 | 0.61 | 1.59 | 0.9537 |
| Summer - BW_6_ vs. Spring - BW_7_ | 0.99 | 0.57 | 1.74 | 0.9789 |
| Summer - BW_7_ vs. Spring - BW_7_ | 1.59 | 1.04 | 2.43 | 0.0323 |
| Autumn - BW1 vs. Spring - BW7 | 1.74 | 1.15 | 2.64 | 0.0092 |
| Autumn - BW2 vs. Spring - BW7 | 1.11 | 0.72 | 1.71 | 0.6414 |
| Autumn - BW3 vs. Spring - BW7 | 0.88 | 0.57 | 1.38 | 0.5831 |
| Autumn - BW4 vs. Spring - BW7 | 0.83 | 0.51 | 1.34 | 0.4456 |
| Autumn - BW5 vs. Spring - BW7 | 1.00 | 0.63 | 1.58 | 0.9984 |
| Autumn - BW6 vs. Spring - BW7 | 1.51 | 0.96 | 2.39 | 0.0772 |
| Autumn - BW7 vs. Spring - BW7 | 0.90 | 0.59 | 1.38 | 0.6366 |
| Winter - BW_1_ vs. Spring - BW_7_ | 1.31 | 0.82 | 2.10 | 0.2667 |
| Winter - BW_2_ vs. Spring - BW_7_ | 1.30 | 0.82 | 2.04 | 0.2639 |
| Winter - BW_3_ vs. Spring - BW_7_ | 1.04 | 0.65 | 1.66 | 0.8664 |
| Winter - BW_4_ vs. Spring - BW_7_ | 0.80 | 0.49 | 1.33 | 0.3893 |
| Winter - BW_5_ vs. Spring - BW_7_ | 0.86 | 0.53 | 1.39 | 0.5323 |
| Winter - BW_6_ vs. Spring - BW_7_ | 0.92 | 0.55 | 1.52 | 0.7323 |
| Winter - BW_7_ vs. Spring - BW_7_ | 0.85 | 0.54 | 1.31 | 0.4557 |
| Summer - BW_2_ vs. Summer - BW_1_ | 0.67 | 0.48 | 0.92 | 0.0121 |
| Summer - BW_3_ vs. Summer - BW_1_ | 0.41 | 0.29 | 0.58 | <.0001 |
| Summer - BW_4_ vs. Summer - BW_1_ | 0.59 | 0.41 | 0.85 | 0.0049 |
| Summer - BW_5_ vs. Summer - BW_1_ | 0.39 | 0.26 | 0.58 | <.0001 |
| Summer - BW_6_ vs. Summer - BW_1_ | 0.39 | 0.24 | 0.64 | 0.0002 |
| Summer - BW_7_ vs. Summer - BW_1_ | 0.63 | 0.45 | 0.88 | 0.0071 |
| Autumn - BW1 vs. Summer - BW1 | 0.69 | 0.50 | 0.95 | 0.0223 |
| Autumn - BW2 vs. Summer - BW1 | 0.44 | 0.31 | 0.62 | <.0001 |
| Autumn - BW3 vs. Summer - BW1 | 0.35 | 0.24 | 0.50 | <.0001 |
| Autumn - BW4 vs. Summer - BW1 | 0.33 | 0.22 | 0.49 | <.0001 |
| Autumn - BW5 vs. Summer - BW1 | 0.39 | 0.27 | 0.58 | <.0001 |
| Autumn - BW6 vs. Summer - BW1 | 0.60 | 0.40 | 0.88 | 0.0099 |
| Autumn - BW7 vs. Summer - BW1 | 0.36 | 0.25 | 0.51 | <.0001 |
| Winter - BW_1_ vs. Summer - BW_1_ | 0.52 | 0.35 | 0.76 | 0.0009 |
| Winter - BW_2_ vs. Summer - BW_1_ | 0.51 | 0.35 | 0.74 | 0.0004 |
| Winter - BW_3_ vs. Summer - BW_1_ | 0.41 | 0.28 | 0.61 | <.0001 |
| Winter - BW_4_ vs. Summer - BW_1_ | 0.32 | 0.21 | 0.49 | <.0001 |
| Winter - BW_5_ vs. Summer - BW_1_ | 0.34 | 0.22 | 0.51 | <.0001 |
| Winter - BW_6_ vs. Summer - BW_1_ | 0.36 | 0.23 | 0.56 | <.0001 |
| Winter - BW_7_ vs. Summer - BW_1_ | 0.33 | 0.23 | 0.48 | <.0001 |
| Summer - BW_3_ vs. Summer - BW_2_ | 0.61 | 0.42 | 0.88 | 0.0085 |
| Summer - BW_4_ vs. Summer - BW_2_ | 0.88 | 0.60 | 1.29 | 0.5216 |
| Summer - BW_5_ vs. Summer - BW_2_ | 0.59 | 0.39 | 0.88 | 0.01 |
| Summer - BW_6_ vs. Summer - BW_2_ | 0.59 | 0.36 | 0.98 | 0.0395 |
| Summer - BW_7_ vs. Summer - BW_2_ | 0.94 | 0.66 | 1.34 | 0.7378 |
| Autumn - BW1 vs. Summer - BW2 | 1.03 | 0.74 | 1.45 | 0.8517 |
| Autumn - BW2 vs. Summer - BW2 | 0.66 | 0.46 | 0.94 | 0.022 |
| Autumn - BW3 vs. Summer - BW2 | 0.52 | 0.36 | 0.76 | 0.0007 |
| Autumn - BW4 vs. Summer - BW2 | 0.49 | 0.32 | 0.75 | 0.0009 |
| Autumn - BW5 vs. Summer - BW2 | 0.59 | 0.40 | 0.88 | 0.0098 |
| Autumn - BW6 vs. Summer - BW2 | 0.90 | 0.60 | 1.34 | 0.5944 |
| Autumn - BW7 vs. Summer - BW2 | 0.54 | 0.37 | 0.77 | 0.0008 |
| Winter - BW_1_ vs. Summer - BW_2_ | 0.78 | 0.52 | 1.16 | 0.2149 |
| Winter - BW_2_ vs. Summer - BW_2_ | 0.77 | 0.52 | 1.13 | 0.178 |
| Winter - BW_3_ vs. Summer - BW_2_ | 0.62 | 0.41 | 0.92 | 0.0192 |
| Winter - BW_4_ vs. Summer - BW_2_ | 0.48 | 0.31 | 0.74 | 0.001 |
| Winter - BW_5_ vs. Summer - BW_2_ | 0.51 | 0.33 | 0.78 | 0.0019 |
| Winter - BW_6_ vs. Summer - BW_2_ | 0.54 | 0.35 | 0.85 | 0.0082 |
| Winter - BW_7_ vs. Summer - BW_2_ | 0.50 | 0.34 | 0.73 | 0.0004 |
| Summer - BW_4_ vs. Summer - BW_3_ | 1.45 | 0.96 | 2.18 | 0.0783 |
| Summer - BW_5_ vs. Summer - BW_3_ | 0.96 | 0.62 | 1.48 | 0.8407 |
| Summer - BW_6_ vs. Summer - BW_3_ | 0.96 | 0.57 | 1.63 | 0.8873 |
| Summer - BW_7_ vs. Summer - BW_3_ | 1.54 | 1.05 | 2.25 | 0.0256 |
| Autumn - BW1 vs. Summer - BW3 | 1.69 | 1.17 | 2.44 | 0.0053 |
| Autumn - BW2 vs. Summer - BW3 | 1.08 | 0.73 | 1.59 | 0.7144 |
| Autumn - BW3 vs. Summer - BW3 | 0.86 | 0.57 | 1.28 | 0.4495 |
| Autumn - BW4 vs. Summer - BW3 | 0.81 | 0.52 | 1.25 | 0.3351 |
| Autumn - BW5 vs. Summer - BW3 | 0.97 | 0.64 | 1.48 | 0.8856 |
| Autumn - BW6 vs. Summer - BW3 | 1.47 | 0.96 | 2.24 | 0.0761 |
| Autumn - BW7 vs. Summer - BW3 | 0.88 | 0.59 | 1.29 | 0.503 |
| Winter - BW_1_ vs. Summer - BW_3_ | 1.27 | 0.83 | 1.95 | 0.2789 |
| Winter - BW_2_ vs. Summer - BW_3_ | 1.26 | 0.83 | 1.90 | 0.2759 |
| Winter - BW_3_ vs. Summer - BW_3_ | 1.01 | 0.66 | 1.55 | 0.9645 |
| Winter - BW_4_ vs. Summer - BW_3_ | 0.78 | 0.49 | 1.24 | 0.2917 |
| Winter - BW_5_ vs. Summer - BW_3_ | 0.83 | 0.53 | 1.30 | 0.4203 |
| Winter - BW_6_ vs. Summer - BW_3_ | 0.89 | 0.55 | 1.43 | 0.6231 |
| Winter - BW_7_ vs. Summer - BW_3_ | 0.82 | 0.55 | 1.23 | 0.3392 |
| Summer - BW_5_ vs. Summer - BW_4_ | 0.66 | 0.43 | 1.03 | 0.0673 |
| Summer - BW_6_ vs. Summer - BW_4_ | 0.67 | 0.39 | 1.13 | 0.1322 |
| Summer - BW_7_ vs. Summer - BW_4_ | 1.07 | 0.73 | 1.57 | 0.7428 |
| Autumn - BW1 vs. Summer - BW4 | 1.17 | 0.79 | 1.73 | 0.4301 |
| Autumn - BW2 vs. Summer - BW4 | 0.74 | 0.50 | 1.12 | 0.1544 |
| Autumn - BW3 vs. Summer - BW4 | 0.59 | 0.39 | 0.90 | 0.0142 |
| Autumn - BW4 vs. Summer - BW4 | 0.56 | 0.35 | 0.88 | 0.012 |
| Autumn - BW5 vs. Summer - BW4 | 0.67 | 0.43 | 1.04 | 0.0737 |
| Autumn - BW6 vs. Summer - BW4 | 1.02 | 0.66 | 1.58 | 0.9448 |
| Autumn - BW7 vs. Summer - BW4 | 0.61 | 0.40 | 0.91 | 0.0156 |
| Winter - BW_1_ vs. Summer - BW_4_ | 0.88 | 0.56 | 1.37 | 0.5673 |
| Winter - BW_2_ vs. Summer - BW_4_ | 0.87 | 0.57 | 1.34 | 0.5245 |
| Winter - BW_3_ vs. Summer - BW_4_ | 0.70 | 0.45 | 1.09 | 0.1149 |
| Winter - BW_4_ vs. Summer - BW_4_ | 0.54 | 0.33 | 0.87 | 0.0118 |
| Winter - BW_5_ vs. Summer - BW_4_ | 0.58 | 0.36 | 0.92 | 0.0199 |
| Winter - BW_6_ vs. Summer - BW_4_ | 0.62 | 0.38 | 1.00 | 0.0505 |
| Winter - BW_7_ vs. Summer - BW_4_ | 0.57 | 0.37 | 0.87 | 0.0085 |
| Summer - BW_6_ vs. Summer - BW_5_ | 1.01 | 0.58 | 1.75 | 0.9816 |
| Summer - BW_7_ vs. Summer - BW_5_ | 1.61 | 1.07 | 2.44 | 0.0236 |
| Autumn - BW1 vs. Summer - BW5 | 1.77 | 1.17 | 2.67 | 0.0069 |
| Autumn - BW2 vs. Summer - BW5 | 1.12 | 0.73 | 1.73 | 0.5926 |
| Autumn - BW3 vs. Summer - BW5 | 0.90 | 0.58 | 1.39 | 0.6232 |
| Autumn - BW4 vs. Summer - BW5 | 0.84 | 0.52 | 1.35 | 0.4776 |
| Autumn - BW5 vs. Summer - BW5 | 1.01 | 0.64 | 1.60 | 0.9534 |
| Autumn - BW6 vs. Summer - BW5 | 1.53 | 0.97 | 2.42 | 0.0666 |
| Autumn - BW7 vs. Summer - BW5 | 0.92 | 0.60 | 1.40 | 0.6828 |
| Winter - BW_1_ vs. Summer - BW_5_ | 1.33 | 0.83 | 2.12 | 0.238 |
| Winter - BW_2_ vs. Summer - BW_5_ | 1.31 | 0.84 | 2.06 | 0.2344 |
| Winter - BW_3_ vs. Summer - BW_5_ | 1.06 | 0.66 | 1.68 | 0.8191 |
| Winter - BW_4_ vs. Summer - BW_5_ | 0.81 | 0.49 | 1.34 | 0.4175 |
| Winter - BW_5_ vs. Summer - BW_5_ | 0.87 | 0.54 | 1.41 | 0.5684 |
| Winter - BW_6_ vs. Summer - BW_5_ | 0.93 | 0.56 | 1.54 | 0.7727 |
| Winter - BW_7_ vs. Summer - BW_5_ | 0.86 | 0.55 | 1.33 | 0.4945 |
| Summer - BW_7_ vs. Summer - BW_6_ | 1.60 | 0.97 | 2.65 | 0.0674 |
| Autumn - BW1 vs. Summer - BW6 | 1.76 | 1.05 | 2.92 | 0.0309 |
| Autumn - BW2 vs. Summer - BW6 | 1.12 | 0.66 | 1.89 | 0.6791 |
| Autumn - BW3 vs. Summer - BW6 | 0.89 | 0.52 | 1.52 | 0.6677 |
| Autumn - BW4 vs. Summer - BW6 | 0.84 | 0.48 | 1.47 | 0.5332 |
| Autumn - BW5 vs. Summer - BW6 | 1.01 | 0.58 | 1.74 | 0.9796 |
| Autumn - BW6 vs. Summer - BW6 | 1.52 | 0.88 | 2.63 | 0.1302 |
| Autumn - BW7 vs. Summer - BW6 | 0.91 | 0.54 | 1.53 | 0.7195 |
| Winter - BW_1_ vs. Summer - BW_6_ | 1.32 | 0.76 | 2.30 | 0.3324 |
| Winter - BW_2_ vs. Summer - BW_6_ | 1.31 | 0.76 | 2.24 | 0.3345 |
| Winter - BW_3_ vs. Summer - BW_6_ | 1.05 | 0.60 | 1.83 | 0.8656 |
| Winter - BW_4_ vs. Summer - BW_6_ | 0.81 | 0.45 | 1.45 | 0.4736 |
| Winter - BW_5_ vs. Summer - BW_6_ | 0.86 | 0.49 | 1.53 | 0.6119 |
| Winter - BW_6_ vs. Summer - BW_6_ | 0.92 | 0.51 | 1.66 | 0.7871 |
| Winter - BW_7_ vs. Summer - BW_6_ | 0.85 | 0.50 | 1.45 | 0.5553 |
| Autumn - BW_1_ vs. Summer - BW_7_ | 1.10 | 0.77 | 1.57 | 0.6138 |
| Autumn - BW_2_ vs. Summer - BW_7_ | 0.70 | 0.48 | 1.02 | 0.0605 |
| Autumn - BW_3_ vs. Summer - BW_7_ | 0.56 | 0.38 | 0.82 | 0.0029 |
| Autumn - BW_4_ vs. Summer - BW_7_ | 0.52 | 0.34 | 0.80 | 0.0029 |
| Autumn - BW_5_ vs. Summer - BW_7_ | 0.63 | 0.42 | 0.94 | 0.0249 |
| Autumn - BW_6_ vs. Summer - BW_7_ | 0.95 | 0.64 | 1.43 | 0.8126 |
| Autumn - BW_7_ vs. Summer - BW_7_ | 0.57 | 0.39 | 0.82 | 0.0026 |
| Winter - BW_1_ vs. Summer - BW_7_ | 0.82 | 0.54 | 1.25 | 0.3635 |
| Winter - BW_2_ vs. Summer - BW_7_ | 0.82 | 0.55 | 1.22 | 0.3184 |
| Winter - BW_3_ vs. Summer - BW_7_ | 0.66 | 0.43 | 0.99 | 0.0464 |
| Winter - BW_4_ vs. Summer - BW_7_ | 0.51 | 0.32 | 0.80 | 0.0032 |
| Winter - BW_5_ vs. Summer - BW_7_ | 0.54 | 0.35 | 0.83 | 0.0055 |
| Winter - BW_6_ vs. Summer - BW_7_ | 0.58 | 0.36 | 0.91 | 0.0185 |
| Winter - BW_7_ vs. Summer - BW_7_ | 0.53 | 0.36 | 0.78 | 0.0013 |
| Autumn - BW_2_ vs. Autumn - BW_1_ | 0.64 | 0.45 | 0.90 | 0.0107 |
| Autumn - BW_3_ vs. Autumn - BW_1_ | 0.51 | 0.35 | 0.73 | 0.0002 |
| Autumn - BW_4_ vs. Autumn - BW_1_ | 0.48 | 0.32 | 0.71 | 0.0003 |
| Autumn - BW_5_ vs. Autumn - BW_1_ | 0.57 | 0.39 | 0.84 | 0.0044 |
| Autumn - BW_6_ vs. Autumn - BW_1_ | 0.87 | 0.59 | 1.28 | 0.4723 |
| Autumn - BW_7_ vs. Autumn - BW_1_ | 0.52 | 0.36 | 0.74 | 0.0002 |
| Winter - BW_1_ vs. Autumn - BW_1_ | 0.75 | 0.50 | 1.12 | 0.1568 |
| Winter - BW_2_ vs. Autumn - BW_1_ | 0.74 | 0.51 | 1.09 | 0.1251 |
| Winter - BW_3_ vs. Autumn - BW_1_ | 0.60 | 0.40 | 0.89 | 0.0114 |
| Winter - BW_4_ vs. Autumn - BW_1_ | 0.46 | 0.30 | 0.72 | 0.0005 |
| Winter - BW_5_ vs. Autumn - BW_1_ | 0.49 | 0.32 | 0.75 | 0.001 |
| Winter - BW_6_ vs. Autumn - BW_1_ | 0.53 | 0.34 | 0.82 | 0.0049 |
| Winter - BW_7_ vs. Autumn - BW_1_ | 0.49 | 0.33 | 0.71 | 0.0002 |
| Autumn - BW_3_ vs. Autumn - BW_2_ | 0.80 | 0.55 | 1.16 | 0.2383 |
| Autumn - BW_4_ vs. Autumn - BW_2_ | 0.75 | 0.49 | 1.14 | 0.1744 |
| Autumn - BW_5_ vs. Autumn - BW_2_ | 0.90 | 0.61 | 1.34 | 0.6081 |
| Autumn - BW_6_ vs. Autumn - BW_2_ | 1.37 | 0.92 | 2.03 | 0.1243 |
| Autumn - BW_7_ vs. Autumn - BW_2_ | 0.81 | 0.56 | 1.18 | 0.2713 |
| Winter - BW_1_ vs. Autumn - BW_2_ | 1.18 | 0.78 | 1.79 | 0.4379 |
| Winter - BW_2_ vs. Autumn - BW_2_ | 1.17 | 0.79 | 1.74 | 0.4411 |
| Winter - BW_3_ vs. Autumn - BW_2_ | 0.94 | 0.62 | 1.42 | 0.7676 |
| Winter - BW_4_ vs. Autumn - BW_2_ | 0.72 | 0.46 | 1.14 | 0.1632 |
| Winter - BW_5_ vs. Autumn - BW_2_ | 0.77 | 0.50 | 1.20 | 0.2492 |
| Winter - BW_6_ vs. Autumn - BW_2_ | 0.83 | 0.52 | 1.31 | 0.4174 |
| Winter - BW_7_ vs. Autumn - BW_2_ | 0.76 | 0.52 | 1.13 | 0.1771 |
| Autumn - BW_4_ vs. Autumn - BW_3_ | 0.94 | 0.61 | 1.44 | 0.7749 |
| Autumn - BW_5_ vs. Autumn - BW_3_ | 1.13 | 0.76 | 1.70 | 0.5487 |
| Autumn - BW_6_ vs. Autumn - BW_3_ | 1.71 | 1.14 | 2.57 | 0.0091 |
| Autumn - BW_7_ vs. Autumn - BW_3_ | 1.02 | 0.70 | 1.49 | 0.912 |
| Winter - BW_1_ vs. Autumn - BW_3_ | 1.48 | 0.96 | 2.27 | 0.0734 |
| Winter - BW_2_ vs. Autumn - BW_3_ | 1.47 | 0.97 | 2.21 | 0.067 |
| Winter - BW_3_ vs. Autumn - BW_3_ | 1.18 | 0.77 | 1.81 | 0.4514 |
| Winter - BW_4_ vs. Autumn - BW_3_ | 0.91 | 0.57 | 1.45 | 0.6837 |
| Winter - BW_5_ vs. Autumn - BW_3_ | 0.97 | 0.62 | 1.52 | 0.8928 |
| Winter - BW_6_ vs. Autumn - BW_3_ | 1.04 | 0.65 | 1.66 | 0.8824 |
| Winter - BW_7_ vs. Autumn - BW_3_ | 0.96 | 0.64 | 1.43 | 0.832 |
| Autumn - BW_5_ vs. Autumn - BW_4_ | 1.20 | 0.78 | 1.87 | 0.4064 |
| Autumn - BW_6_ vs. Autumn - BW_4_ | 1.82 | 1.18 | 2.82 | 0.0072 |
| Autumn - BW_7_ vs. Autumn - BW_4_ | 1.09 | 0.72 | 1.64 | 0.6928 |
| Winter - BW_1_ vs. Autumn - BW_4_ | 1.58 | 0.99 | 2.51 | 0.057 |
| Winter - BW_2_ vs. Autumn - BW_4_ | 1.56 | 1.00 | 2.45 | 0.0522 |
| Winter - BW_3_ vs. Autumn - BW_4_ | 1.25 | 0.79 | 2.00 | 0.3401 |
| Winter - BW_4_ vs. Autumn - BW_4_ | 0.97 | 0.59 | 1.59 | 0.8924 |
| Winter - BW_5_ vs. Autumn - BW_4_ | 1.03 | 0.64 | 1.67 | 0.899 |
| Winter - BW_6_ vs. Autumn - BW_4_ | 1.10 | 0.67 | 1.83 | 0.7046 |
| Winter - BW_7_ vs. Autumn - BW_4_ | 1.02 | 0.66 | 1.58 | 0.9343 |
| Autumn - BW_6_ vs. Autumn - BW_5_ | 1.51 | 1.00 | 2.29 | 0.0493 |
| Autumn - BW_7_ vs. Autumn - BW_5_ | 0.90 | 0.61 | 1.33 | 0.6056 |
| Winter - BW_1_ vs. Autumn - BW_5_ | 1.31 | 0.83 | 2.05 | 0.2427 |
| Winter - BW_2_ vs. Autumn - BW_5_ | 1.30 | 0.84 | 2.00 | 0.2381 |
| Winter - BW_3_ vs. Autumn - BW_5_ | 1.04 | 0.67 | 1.63 | 0.8584 |
| Winter - BW_4_ vs. Autumn - BW_5_ | 0.80 | 0.50 | 1.30 | 0.3707 |
| Winter - BW_5_ vs. Autumn - BW_5_ | 0.86 | 0.54 | 1.37 | 0.5156 |
| Winter - BW_6_ vs. Autumn - BW_5_ | 0.92 | 0.56 | 1.49 | 0.7235 |
| Winter - BW_7_ vs. Autumn - BW_5_ | 0.85 | 0.56 | 1.29 | 0.4352 |
| Autumn - BW_7_ vs. Autumn - BW_6_ | 0.60 | 0.41 | 0.88 | 0.0087 |
| Winter - BW_1_ vs. Autumn - BW_6_ | 0.86 | 0.55 | 1.36 | 0.5279 |
| Winter - BW_2_ vs. Autumn - BW_6_ | 0.86 | 0.56 | 1.32 | 0.4846 |
| Winter - BW_3_ vs. Autumn - BW_6_ | 0.69 | 0.44 | 1.08 | 0.1025 |
| Winter - BW_4_ vs. Autumn - BW_6_ | 0.53 | 0.33 | 0.86 | 0.0101 |
| Winter - BW_5_ vs. Autumn - BW_6_ | 0.57 | 0.36 | 0.90 | 0.0168 |
| Winter - BW_6_ vs. Autumn - BW_6_ | 0.61 | 0.37 | 0.99 | 0.0435 |
| Winter - BW_7_ vs. Autumn - BW_6_ | 0.56 | 0.37 | 0.85 | 0.0065 |
| Winter - BW_1_ vs. Autumn - BW_7_ | 1.45 | 0.95 | 2.21 | 0.0853 |
| Winter - BW_2_ vs. Autumn - BW_7_ | 1.44 | 0.96 | 2.15 | 0.0772 |
| Winter - BW_3_ vs. Autumn - BW_7_ | 1.15 | 0.76 | 1.75 | 0.5008 |
| Winter - BW_4_ vs. Autumn - BW_7_ | 0.89 | 0.56 | 1.40 | 0.611 |
| Winter - BW_5_ vs. Autumn - BW_7_ | 0.95 | 0.61 | 1.47 | 0.8148 |
| Winter - BW_6_ vs. Autumn - BW_7_ | 1.01 | 0.64 | 1.60 | 0.9509 |
| Winter - BW_7_ vs. Autumn - BW_7_ | 0.94 | 0.64 | 1.38 | 0.7403 |
| Winter - BW_2_ vs. Winter - BW_1_ | 0.99 | 0.64 | 1.53 | 0.9684 |
| Winter - BW_3_ vs. Winter - BW_1_ | 0.80 | 0.51 | 1.25 | 0.3224 |
| Winter - BW_4_ vs. Winter - BW_1_ | 0.61 | 0.38 | 1.00 | 0.0498 |
| Winter - BW_5_ vs. Winter - BW_1_ | 0.66 | 0.41 | 1.05 | 0.0793 |
| Winter - BW_6_ vs. Winter - BW_1_ | 0.70 | 0.43 | 1.15 | 0.1589 |
| Winter - BW_7_ vs. Winter - BW_1_ | 0.65 | 0.42 | 1.00 | 0.0476 |
| Winter - BW_3_ vs. Winter - BW_2_ | 0.80 | 0.53 | 1.23 | 0.3133 |
| Winter - BW_4_ vs. Winter - BW_2_ | 0.62 | 0.39 | 0.99 | 0.043 |
| Winter - BW_5_ vs. Winter - BW_2_ | 0.66 | 0.42 | 1.03 | 0.0689 |
| Winter - BW_6_ vs. Winter - BW_2_ | 0.71 | 0.44 | 1.13 | 0.1485 |
| Winter - BW_7_ vs. Winter - BW_2_ | 0.65 | 0.44 | 0.98 | 0.0375 |
| Winter - BW_4_ vs. Winter - BW_3_ | 0.77 | 0.48 | 1.24 | 0.2854 |
| Winter - BW_5_ vs. Winter - BW_3_ | 0.82 | 0.52 | 1.30 | 0.4055 |
| Winter - BW_6_ vs. Winter - BW_3_ | 0.88 | 0.54 | 1.43 | 0.6021 |
| Winter - BW_7_ vs. Winter - BW_3_ | 0.81 | 0.54 | 1.23 | 0.3262 |
| Winter - BW_5_ vs. Winter - BW_4_ | 1.07 | 0.65 | 1.75 | 0.7945 |
| Winter - BW_6_ vs. Winter - BW_4_ | 1.14 | 0.68 | 1.92 | 0.6165 |
| Winter - BW_7_ vs. Winter - BW_4_ | 1.05 | 0.67 | 1.66 | 0.8189 |
| Winter - BW_6_ vs. Winter - BW_5_ | 1.07 | 0.65 | 1.76 | 0.7941 |
| Winter - BW_7_ vs. Winter - BW_5_ | 0.99 | 0.64 | 1.52 | 0.9538 |
| Winter - BW_7_ vs. Winter - BW_6_ | 0.92 | 0.59 | 1.46 | 0.7336 |
| **Parity× Twin birth** |  |  |  |  |
| Parity_1_ - TWN vs. Parity_1_ - ST | 66.86 | 51.49 | 86.82 | <.0001 |
| Parity_2_ - ST vs. Parity_1_ - ST | 1.22 | 1.03 | 1.46 | 0.0246 |
| Parity_2_ - TWN vs. Parity_1_ - ST | 192.88 | 143.37 | 259.48 | <.0001 |
| Parity_3_ - ST vs. Parity_1_ - ST | 1.14 | 0.92 | 1.42 | 0.2362 |
| Parity_3_ - TWN vs. Parity_1_ - ST | 188.98 | 133.45 | 267.62 | <.0001 |
| Parity_≥4_ - ST vs. Parity_1_ - ST | 0.75 | 0.59 | 0.96 | 0.0201 |
| Parity_≥4_ - TWN vs. Parity_1_ - ST | 399.36 | 256.42 | 621.98 | <.0001 |
| Parity_2_ - ST vs. Parity_1_ - TWN | 0.02 | 0.01 | 0.02 | <.0001 |
| Parity_2_ - TWN vs. Parity_1_ - TWN | 2.89 | 2.15 | 3.87 | <.0001 |
| Parity_3_ - ST vs. Parity_1_ - TWN | 0.02 | 0.01 | 0.02 | <.0001 |
| Parity_3_ - TWN vs. Parity_1_ - TWN | 2.83 | 1.99 | 4.02 | <.0001 |
| Parity_≥4_ - ST vs. Parity_1_ - TWN | 0.01 | 0.01 | 0.02 | <.0001 |
| Parity_≥4_ - TWN vs. Parity_1_ - TWN | 5.97 | 3.83 | 9.33 | <.0001 |
| Parity_2_ - TWN vs. Parity_2_ - ST | 157.80 | 115.43 | 215.73 | <.0001 |
| Parity_3_ - ST vs. Parity_2_ - ST | 0.93 | 0.73 | 1.19 | 0.5687 |
| Parity_3_ - TWN vs. Parity_2_ - ST | 154.61 | 107.71 | 221.94 | <.0001 |
| Parity_≥4_ - ST vs. Parity_2_ - ST | 0.62 | 0.48 | 0.80 | 0.0003 |
| Parity_≥4_ - TWN vs. Parity_2_ - ST | 326.73 | 207.68 | 514.01 | <.0001 |
| Parity_3_ - ST vs. Parity_2_ - TWN | 0.01 | 0.00 | 0.01 | <.0001 |
| Parity_3_ - TWN vs. Parity_2_ - TWN | 0.98 | 0.67 | 1.44 | 0.9174 |
| Parity_≥4_ - ST vs. Parity_2_ - TWN | 0.00 | 0.00 | 0.01 | <.0001 |
| Parity_≥4_ - TWN vs. Parity_2_ - TWN | 2.07 | 1.29 | 3.32 | 0.0024 |
| Parity_3_ - TWN vs. Parity_3_ - ST | 165.82 | 113.16 | 242.98 | <.0001 |
| Parity_≥4_ - ST vs. Parity_3_ - ST | 0.66 | 0.50 | 0.88 | 0.0047 |
| Parity_≥4_ - TWN vs. Parity_3_ - ST | 350.41 | 219.07 | 560.50 | <.0001 |
| Parity_≥4_ - ST vs. Parity_3_ - TWN | 0.00 | 0.00 | 0.01 | <.0001 |
| Parity_≥4_ - TWN vs. Parity_3_ - TWN | 2.11 | 1.27 | 3.51 | 0.0038 |
| Parity_≥4_ - TWN vs. Parity_≥4_ - ST | 529.73 | 328.26 | 854.86 | <.0001 |
| **Dry period length× Twin birth** |  |  |  |  |
| DLP_1_ - TWN vs. DLP_1_ - ST | 195.26 | 152.47 | 250.07 | <.0001 |
| DLP_2_ - ST vs. DLP_1_ - ST | 1.56 | 1.34 | 1.82 | <.0001 |
| DLP_2_ - TWN vs. DLP_1_ - ST | 167.69 | 130.46 | 215.55 | <.0001 |
| DLP_3_ - ST vs. DLP_1_ - ST | 1.34 | 1.06 | 1.68 | 0.0143 |
| DLP_3_ - TWN vs. DLP_1_ - ST | 212.74 | 146.24 | 309.48 | <.0001 |
| DLP_≥4_ - ST vs. DLP_1_ - ST | 0.98 | 0.72 | 1.32 | 0.8798 |
| DLP_≥4_ - TWN vs. DLP_1_- ST | 271.24 | 154.09 | 477.46 | <.0001 |
| DLP_2_ - ST vs. DLP_1_ - TWN | 0.01 | 0.01 | 0.01 | <.0001 |
| DLP_2_ - TWN vs. DLP_1_ - TWN | 0.86 | 0.67 | 1.11 | 0.2434 |
| DLP_3_ - ST vs. DLP_1_ - TWN | 0.01 | 0.01 | 0.01 | <.0001 |
| DLP_3_ - TWN vs. DLP_1_ - TWN | 1.09 | 0.74 | 1.60 | 0.6638 |
| DLP_≥4_ - ST vs. DLP_1_ - TWN | 0.01 | 0.00 | 0.01 | <.0001 |
| DLP_≥4_ - TWN vs. DLP_1_ - TWN | 1.39 | 0.77 | 2.50 | 0.2726 |
| DLP_2_ - TWN vs. DLP_2_ - ST | 107.28 | 83.54 | 137.77 | <.0001 |
| DLP_3_ - ST vs. DLP_2_ - ST | 0.85 | 0.68 | 1.07 | 0.1737 |
| DLP_3_ - TWN vs. DLP_2_ - ST | 136.10 | 93.71 | 197.65 | <.0001 |
| DLP_≥4_ - ST vs. DLP_2_ - ST | 0.63 | 0.46 | 0.85 | 0.0023 |
| DLP_≥4_ - TWN vs. DLP_2_ - ST | 173.53 | 98.56 | 305.52 | <.0001 |
| DLP_3_ - ST vs. DLP_2_ - TWN | 0.01 | 0.01 | 0.01 | <.0001 |
| DLP_3_ - TWN vs. DLP_2_ - TWN | 1.27 | 0.86 | 1.88 | 0.2348 |
| DLP_≥4_ - ST vs. DLP_2_ - TWN | 0.01 | 0.00 | 0.01 | <.0001 |
| DLP_≥4_ - TWN vs. DLP_2_ - TWN | 1.62 | 0.90 | 2.90 | 0.1071 |
| DLP_3_ - TWN vs. DLP_3_ - ST | 159.34 | 106.40 | 238.61 | <.0001 |
| DLP_≥4_ - ST vs. DLP_3_ - ST | 0.73 | 0.52 | 1.03 | 0.0708 |
| DLP_≥4_ - TWN vs. DLP_3_ - ST | 203.16 | 112.98 | 365.33 | <.0001 |
| DLP_≥4_ - ST vs. DLP_3_ - TWN | 0.01 | 0.00 | 0.01 | <.0001 |
| DLP_≥4_ - TWN vs. DLP_3_ - TWN | 1.28 | 0.67 | 2.44 | 0.4619 |
| DLP_≥4_ - TWN vs. DLP_≥4_ - ST | 277.66 | 149.69 | 515.07 | <.0001 |
| § ST= Single; TWN = twin, SB: stillbirth, BW= calf birth weight  BW1-7: 1 = ≤ 35 kg, 2 = 35.1-38 kg, 3 = 38.1- 40 kg, 4 = 40.1 -42 kg, 5 = 42.1-44 kg, 6 = 44.1- 46 kg, 7 = > 46 kg.  DLP_i_ = dry period length (i:1-4; < 45d; 46-60 d; 61 -75 d and > 75 d) | | | | |

**Supplemental Table S3D:** Calving number for each item was included in the significant interaction between cow-level risk factors associated with stillbirth in the binary logistic regression model of the generalized linear mixed model in Holstein dairy cows (n = 51,405).

| **Item** | **Calving No.** |
| --- | --- |
| Spring - ST | 10716 |
| Spring - TWN | 15050 |
| Summer - ST | 13050 |
| Summer - TWN | 10775 |
| Autumn - ST | 398 |
| Autumn - TWN | 637 |
| Winter - ST | 448 |
| Winter - TWN | 331 |
| Spring - BW_1_ | 1540 |
| Spring - BW_2_ | 1899 |
| Spring - BW_3_ | 1796 |
| Spring - BW_4_ | 1552 |
| Spring - BW_5_ | 1463 |
| Spring - BW_6_ | 1089 |
| Spring - BW_7_ | 1775 |
| Summer - BW_1_ | 2356 |
| Summer - BW_2_ | 2696 |
| Summer - BW_3_ | 2610 |
| Summer - BW_4_ | 2218 |
| Summer - BW_5_ | 2066 |
| Summer - BW_6_ | 1318 |
| Summer - BW_7_ | 2423 |
| Autumn - BW1 | 1748 |
| Autumn - BW2 | 2149 |
| Autumn - BW3 | 2287 |
| Autumn - BW4 | 1849 |
| Autumn - BW5 | 1860 |
| Autumn - BW6 | 1334 |
| Autumn - BW7 | 2271 |
| Winter - BW_1_ | 1162 |
| Winter - BW_2_ | 1680 |
| Winter - BW_3_ | 1642 |
| Winter - BW_4_ | 1473 |
| Winter - BW_5_ | 1696 |
| Winter - BW_6_ | 1219 |
| Winter - BW_7_ | 2234 |
| Parity_1_ - ST | 30256 |
| Parity_2_ - ST | 8510 |
| Parity_3_ - ST | 5101 |
| Parity_≥4_ - ST | 5724 |
| Parity_1_ - TWN | 837 |
| Parity_2_ - TWN | 434 |
| Parity_3_ - TWN | 277 |
| Parity_≥4_ - TWN | 266 |
| DLP_1_ - ST | 25521 |
| DLP_2_ - ST | 15484 |
| DLP_3_ - ST | 5734 |
| DLP_≥4_ - ST | 2852 |
| DLP_1_ - TWN | 895 |
| DLP_2_ - TWN | 572 |
| DLP_3_ - TWN | 223 |
| DLP_≥4_ - TWN | 124 |

§ ST= Single; TWN = twin, SB: stillbirth, BW= calf birth weight

BW1-7: 1 = ≤ 35 kg, 2 = 35.1-38 kg, 3 = 38.1- 40 kg, 4 = 40.1 -42 kg, 5 = 42.1-44 kg, 6 = 44.1- 46 kg, 7 = > 46 kg.

DLP_i_ = dry period length (i:1-4; < 45d; 46-60 d; 61 -75 d and > 75 d).

**Supplemental Table S4:** Estimated odds ratios and 95% confidence levels (CI) of selected blood macro-minerals at parturition included in the binary logistic regression model of the generalized linear mixed model for stillbirth incidence in Holstein dairy cows (n = 1,311)

| Variable | Odds ratio (95% CI) |
| --- | --- |
| Calcium (mg/dl) |  |
| Hypo (≤ 8) | Referent |
| Normal (> 8.1) | 0.66 (0.51-0.98) |
| Phosphorus (mg/dl) |  |
| Hypo (≤ 4) | Referent |
| Normal (> 4.1) | 0.98 (0.86-1.09) |
| Magnesium (mg/dl) |  |
| Hypo (≤ 2.5) | Referent |
| Normal (> 2.51) | 0.94 (0.86-1.21) |
